# PROTOCOL: The effects of empowerment‐based nutrition interventions on the nutritional status of adolescent girls in low‐ and middle‐income countries

**DOI:** 10.1002/cl2.1042

**Published:** 2019-09-05

**Authors:** Alison Y. Riddle, Cynthia M. Kroeger, Abigail K. Ramage, Zulfiqar A. Bhutta, Elizabeth Kristjansson, Carol Vlassoff, Monica Taljaard, Becky Skidmore, Vivian Welch, George A. Wells

**Affiliations:** ^1^ School of Epidemiology and Public Health University of Ottawa Ottawa Ontario Canada; ^2^ School of Pharmacy, Faculty of Medicine and Health University of Sydney Camperdown NSW Australia; ^3^ Independent consultant Cambridge England; ^4^ Centre for Global Child Health Hospital for Sick Children Toronto Ontario Canada; ^5^ School of Psychology University of Ottawa Ottawa Ontario Canada; ^6^ Ottawa Hospital Research Institute Ottawa Ontario Canada; ^7^ Independent Information Specialist Ottawa Canada; ^8^ The Campbell Collaboration Ottawa Ontario Canada; ^9^ Cardiovascular Research Methods Centre University of Ottawa Heart Institute Ottawa Ontario Canada

## 1 Background

### 1.1 The problem, condition or issue

Adolescents (10 to 19 years) currently represent the largest global generation of young people in our collective history (United Nations, 2015). The regions of Africa, Asia, Latin America, and the Caribbean are the home of 1.1 billion young persons (United Nations Department of Economic and Social Affairs Population Division, 2017). In sub‐Saharan Africa, people below the age of 25 make up 62% of the population, with only marginal declines predicted through 2050 (United Nations Department of Economic and Social Affairs Population Division, 2019). The working age population (25 to 64 years) in sub‐Saharan Africa, Oceania, and parts of Asia, Latin America, and the Caribbean is growing faster than all other age groups (United Nations Department of Economic and Social Affairs Population Division, 2019). Ensuring the health and well‐being of adolescents who will fill the ranks of the world's working age population will help to propel global economic growth and development (Patton et al., 2016).

Adolescence is a period of significant physiological change that includes marked skeletal growth, increased bone mass, and fundamental neurological development (Das et al., 2017; Patton et al., 2016). Proper nutrition during adolescence is crucial for optimal growth and development and helps to prepare adolescents for adulthood. However, many adolescents face challenges in achieving optimal dietary intake, especially in low‐ and middle‐income countries (LMICs) where the majority of adolescents reside (WHO, 2014). Iron‐deficiency anemia affects 430.7 million (24%) adolescents, with 77% of adolescents living with anaemia in multiburden countries where communicable, maternal, and nutritional conditions contribute to 2,500 disability adjusted life‐years (DALYs) or more per 100,000 adolescents (Azzopardi et al., 2019). The prevalence of anaemia is consistently higher for females than males, and is more than 50% for females in Bhutan, Yemen, India, and Burkina Faso in 2016 (Azzopardi et al., 2019). Mean BMI (body mass index) and the prevalence of obesity are also rising among children and adolescents globally. The percentage of adolescents who are overweight or obese was 324.1 million in 2016—an increase of 176.9 million from 1990 (Azzopardi et al., 2019). The median prevalence of overweight and obesity among girls is highest in the Eastern Mediterranean region (28%), followed by the Western Pacific (25%), the Americas (25%), and Europe (21%; Akseer, Al‐Gashm, Mehta, Mokdad, & Bhutta, 2017). Yet more children and adolescents are moderately or severely underweight globally, with the largest burden of underweight found in South Asia and central, east and west Africa (Abarca‐Gómez et al., 2017).

The social, economic, and cultural conditions in which an adolescent matures can significantly shape their health and development, including their nutrition. The social determinants of health are the conditions in which people are born, grow, develop, live, work, and age (Viner et al., 2012). Social inequities, particularly those related to poverty and gender, can heavily influence adolescent health and well‐being (Patton et al., 2016). Adolescence is a time when gender roles and norms become more heavily engrained, affecting how adolescents interact with and experience the world. The gendered experience of adolescence can vary depending on the context, as Kabeer writes: “In many LMICs, the gendered norms embedded in local structures of patriarchy come into play in heightened ways during adolescence, restricting the agency, opportunities, aspirations and social networks of young girls to a far greater extent than boys” (Kabeer, 2018). Where considerable gender inequities exist, women and girls are more likely to suffer from nutritional deficiencies than men and boys (Elder, 2003). Gender inequities can limit access to an adequate diet and lead to early marriage and high fertility rates, putting adolescent girls at greater risk of nutrient deficiencies as well as poor pregnancy and birth outcomes (Akseer et al., 2017; Bhutta et al., 2013). Numerous studies have identified a significant association between women's empowerment and women's and children's nutritional status (Alaofè, Zhu, Burney, Naylor, & Douglas, 2017; Bhagowalia, Menon, Quisumbing, & Soundararajan, 2012; Cunningham, Ruel, Ferguson, & Uauy, 2015; Na, Jennings, Talegawkar, & Ahmed, 2015; Pratley, 2016; Sinharoy et al., 2018; Smith, Ramakrishnan, Ndiaye, Haddad, & Martorell, 2003; Taukobong et al., 2016).

### 1.2 The intervention

The empowerment of women and girls has been identified as way of improving the nutrition sensitivity of interventions, in addition to achieving scale and increased coverage of at‐risk households and individuals (Ruel & Alderman, 2013). There is growing interest in assessing the impact of empowerment interventions on women's empowerment as well as its instrumental value in improving development outcomes, including women's and children's health and nutrition. The objective of this is review is to build on the existing literature base to examine the effects of integrating women's empowerment strategies in nutrition interventions to improve the nutritional status of adolescent girls’ in low‐ and middle‐income countries.

### 1.3 Defining women's empowerment and its related components

There are various definitions of women's empowerment, illustrating the complexity of the construct (Alsop & Heinsohn, 2005; Batliwala, 1994; Kabeer, 1999; Lee‐Rife, 2010; Malhotra, Schuler, & Boender, 2002; G. Sen & Batliwala, 2000; A. Sen, 1985a). A commonly used definition is that of Naila Kabeer. She defines empowerment as “the expansion in people's ability to make strategic life choices in a context where this ability was previously denied to them” (Kabeer, 2001). Kabeer identifies three interdependent components to women's empowerment. The first is agency, or “the ability to define one's goals and act upon them” (Kabeer, 2001). Alsop and Heinsohn (2005) define agency as “the ability to make meaningful choices.” Similarly, A. Sen (1985b) describes agency as “what a person is free to do and achieve in pursuit of whatever goals or values he or she regards as important.” Central to these definitions is (a) the availability of alternatives from which to choose, (b) the individual is aware of the alternatives available to them and (c) the individual desires to make a choice (Alsop & Heinsohn, 2005; Kabeer, 2001). Agency is often described in terms of decision‐making power, but it can also reflect an ability to bargain, negotiate, influence, resist, or manipulate (Kabeer, 1999).

Kabeer (2001) identifies the second component of empowerment, *resources*, as a precondition for the ability to exercise choice (agency). Resources can be material, human or social. Alsop and Heinsohn (2005) refer to this component as *opportunity structure*, or “the formal and informal contexts within which actors operate.” We will use the term “opportunity structure” to represent this second component for the purposes of this review. A supportive opportunity structure will enable an individual to make their choices a reality. For example, an adolescent girl may desire to stay in school until graduation, but she may be prevented from doing so if her father prefers her to be married.

The final component is *achievements*. Agency and opportunity structure interact to result in the achievement of an individual's desired outcomes. A. Sen (1985b) refers to this interaction as capabilities, or the potential that people have for living the lives they want. Notably, chosen actions that lead to undesired outcomes do not signify a high degree of empowerment. For example, the decision to participate in a microcredit programme may increase a woman's access to financial resources, but if her husband controls how those resources are spent, the desired outcome of financial autonomy would not be achieved.

Empowerment can take place in different dimensions of women's lives. A high degree of empowerment in one area does not automatically translate into empowerment in other areas. For instance, an adolescent girl may be able to attend school (an indicator of social empowerment) but she may be denied the right to access health care without her guardian's permission (an indicator of household or intrafamilial empowerment). There are six conceptual definitions of empowerment commonly used in the literature (Malhotra et al., 2002; Pratley & Sandberg, 2018). Below we briefly describe each and provide indicators at the individual level.

- *Economic*: Access and claims to material resources. Examples of individual‐level indicators include control over one's income, access to the labour market, having a bank account, etc.
- *Political*: Inclusion in political processes and the ability to self‐organise. Example indicators are the ability to organise and participate in a women's advocacy group or women's membership on a local council.
- *Legal*: Women's rights as codified in law. Example indicators are the ability to own property and the right to vote.
- *Socio‐cultural*: The type and quality of relationships with other people and groups outside of the household, often highly influenced by society’ gender norms. Examples include women's freedom of movement and society's commitment to girls’ education.
- *Intrafamilial*: The social hierarchy and dynamics within the household. Examples are women's role in household decision‐making or their sexual autonomy.
- *Psychological*: An individual's belief that they can achieve their goals. An example is an individual's perception of their own self‐efficacy. This dimension is closely related to the concept of agency.

The gender equality challenges and the relevance of different empowerment dimensions will vary by context. For example, socio‐cultural norms in South Asia can restrict women's ability to leave home without a male relative or chaperone. In sub‐Saharan Africa, migrant labour among men is common, giving women greater levels of independence, if not actual autonomy.

### 1.4 Operationalizing women's empowerment

The operationalization of women's empowerment in health and development programmes in recent years has largely focused on improving access to resources, assets, and services. Interventions that have been assessed include women's self‐help groups (often with an economic focus; Atteraya, Gnawali, & Palley, 2016; Brody et al., 2015; Kabeer, 2018; Kumar et al., 2018; Lahiri‐Dutt & Samanta, 2006; Vollmer, Khan, Ngoc Tu, Pasha, & Sahoo, 2017), cash transfer programmes (Adato, de la Briere, Mindek, & Quisumbing, 2000; Bonilla et al., 2017; Molyneux, 2008; Tiwari et al., 2016; van den Bold, Quisumbing, & Gillespie, 2013), microcredit programmes (Lahiri‐Dutt & Samanta, 2006; Mahmud, 2003; Naser & Crowther, 2016; Swain & Wallentin, 2009; Vaessen et al., 2014; van Rooyen, Stewart, & de Wet, 2012), agriculture programmes (Olney et al., 2016; van den Bold et al., 2015), and programmes to strengthen inheritance and property rights (Allendorf, 2007; Mishra & Sam, 2016; Peterman, 2011).

Shankar, Sundar, and Smith (2019) have written about the gap in focusing specifically on agency‐based interventions, that include “designing spaces that allow individuals to self‐define their goals in life areas such as work, relationships, sexuality, spirituality, or financial security.” Similarly, Cornwall has highlighted the neglect of building “critical consciousness” as part of women's empowerment interventions in favour of a focus on resources (Cornwall, 2016). We will build on their work by assessing the effectiveness of nutrition interventions that include activities to foster agency as well as creating a supportive opportunity structure (including access to resources) as a way to empower adolescent girls and improve their nutritional status. Further, we will summarise the contextual and implementation factors that contribute to the success or failure of these interventions.

### 1.5 Empowerment‐based nutrition interventions

This review will summarise the evidence concerning the effectiveness of *empowerment‐based* nutrition interventions for adolescent girls in low‐ and middle‐income countries. In other words, we will assess the effectives of nutrition interventions that include activities to (a) foster adolescent girls’ *agency* and (b) create a supportive *opportunity structure* for adolescent girls’ empowerment.

For the purposes of this review, we define nutrition interventions as interventions intended to improve dietary intake among adolescent girls by promoting a healthy diet or providing additional micronutrients through fortification or targeted supplementation (World Health Organisation [WHO], 2018a). Examples of eligible nutrition interventions are micronutrient supplementation (iron, folic acid, vitamins A, D, etc.), food supplementation programmes (e.g., school feeding), and nutrition education or counselling.

To be empowerment‐based, the intervention must include activities to support adolescent girls’ agency and activities to create a supportive opportunity structure for adolescent girls’ empowerment. An intervention that fosters agency will include activities designed to increase adolescent girls’ motivations and abilities to make informed decisions by providing spaces for self‐reflection and identification of important life areas (Shankar et al., 2019). Such interventions enable adolescent girls’ active and meaningful participation in decision‐making, instil a sense of self‐efficacy, and increase self‐esteem and motivation to make a positive change in pursuit of strategic life goals. Examples of activities that foster agency are life skills training programmes, mentorship programmes, counselling programmes, and other programmes that create “safe spaces” for adolescent girls or equip adolescent girls to make informed strategic life decisions. Interventions can be delivered one‐on‐one or in a group setting and can take place in a variety of settings include at home, at school and in the community.

The intervention must also include activities to build a supportive opportunity structure. Such activities aim to alter the constraining political, economic, socio‐cultural, interpersonal, and/or legal structures (informal or formal) at the household, community, or broader societal levels, as necessary, to support adolescent girls to exercise agency (Alsop & Heinsohn, 2005; Malhotra et al., 2002). This includes access to and control over resources. The type of activities that are undertaken to create a supportive opportunity structure will vary by context, thus we cannot provide an exhaustive list. Instead, we have attempted to categorize activities by type according to the dimension of empowerment they seek to redress. They are:

- *Economic*: Economic activities aim to increase adolescent girls’ access to and control over financial and material resources. These include microcredit programmes, cash transfer programmes, agriculture programmes, homestead or community gardening programmes, and savings and loan programmes.
- *Socio‐cultural*: Socio‐cultural activities aim to redress discriminatory gender norms, customs and practices that restrict adolescent girls’ ability to exercise agency, most often at the household and community level. Examples include activities to prevent child marriage, programmes to improve freedom of movement, male or in‐law engagement strategies to reduce intrahousehold food discrimination, and programmes to support adolescent girls’ completion of secondary education.
- *Legal*: Legal activities aim to establish laws meant to prevent gender‐based discrimination and protect adolescent girls’ rights. This can include adolescent girls’ rights to education, family planning, employment or inheritance.

Table 1 provides examples of how the three different intervention components can combine to create an eligible intervention study. Each intervention must have as its main activity a nutrition intervention aimed to improve dietary intake among adolescent girls. In addition, an eligible intervention must include agency‐related activities and opportunity structure activities.

**Table 1:** Examples of eligible intervention studies that include all three intervention components

	Nutrition		Agency		Opportunity structure		
Study	Promoting a healthy diet	Micronutrient supplementation or fortification	Individual	Group	Economic	Socio‐cultural	Legal
Study 1	X		X		X		
Study 2		X		X	X	X	
Study 3	X	X		X			X

An example of a primary study that may be included in this review is the Adolescent Girls Empowerment Programme in Zambia (Hewett et al., 2017). The 2‐year, multiarm cluster randomised controlled trial assessed the impact of an “asset‐building framework” intervention on the empowerment and health status of girls aged 10–19 years. The intervention provided nutrition education using participatory methods (nutrition‐specific component), alongside a weekly mentor‐led girls groups meeting covering health, life skills and financial education (agency component), and a health voucher and savings account programme (opportunity structure component). The study assessed a series of anthropometric measures and anaemia status, as well as empowerment outcomes between the intervention and control groups.

### 1.6 How the intervention might work

We hypothesise that including empowerment‐related activities in a nutrition intervention will mediate the impact of underlying gender inequities that contribute to poorer health outcomes for adolescent girls and will result in greater nutritional gains compared with programmes that do not promote women's empowerment.

Our logic model is presented in Appendix A. The logic model depicts the causal pathways from the implementation of an empowerment‐based nutrition intervention to improved nutrition outcomes for adolescent girls. The development of the logic model was informed by a review of existing models and conceptual frameworks on nutrition (Black et al., 2013; Kumar et al., 2018; Salam, Das, Lassi, & Bhutta, 2016; UNICEF, 2015; WHO, 2018b) and women's empowerment (Alsop & Heinsohn, 2005; Kabeer, 1999; Kumar et al., 2018; Malhotra et al., 2002; A. Sen, 1985a; van den Bold et al., 2013; Whitehead et al., 2016).

The model begins with the proposed *three elements* of an empowerment‐based nutrition intervention:

1. Activities to improve dietary intake
2. Activities to foster agency
3. Activities to build a supportive opportunity structure

In the *short term*, the elements are hypothesised to lead to an expansion in an adolescent girls’ awareness of the choices available to her to improve her nutritional status, and to increase her motivation to act. The nutrition‐related activities will improve her awareness, knowledge and skills for the adoption of a healthy diet and positive health behaviours. The agency‐related activities complement the nutrition‐related knowledge she has acquired to increase her awareness and motivation to make an informed choice to improve her health and nutrition. A supportive opportunity structure will enable her to move toward action based on her choices. For example, a school‐based micronutrient supplementation programme for adolescent girls that includes a peer support programme to build girls’ nutrition‐related knowledge and self‐confidence (agency) and a sensitisation programme for parents and teachers to the importance of supplementation for adolescent girls (opportunity structure) will increase a girl's knowledge of the importance of supplementation for her health and well‐being, increase her motivation to participate in the supplementation programme, and create an enabling environment that will provide the necessary resources and supports for her to participate in the programme.

In the *intermediate term*, the intervention is expected to empower adolescent girls by increasing their decision‐making power, improving their access to and control over resources (human, capital, social), and contribute to the protection and promotion of their human rights, such as the right to health care, education, freedom of movement, freedom from violence so forth. Returning to our illustrative example, an adolescent girl will have more decision‐making power because she has acquired the information that she needs to make an informed decision, she has the confidence to make a decision, and she has the support from parents and teachers to enable her decision‐making. Her access to resources is also theoretically improved with the support of the adults in her life. Finally, the sensitisation of parents and teachers should help to prevent any potential restrictions on her rights that may impede her ability to participate in the programme.

Moving down the results chain, the empowerment‐related outcomes are hypothesised to feed into improved health and nutrition behaviours, improved access to a nutritious diet, and improved access to essential health services. In the case of our example, an intermediate outcome would be adolescent girls’ taking micronutrient supplements consistently because they have decided it is important to them, and their environment supports them to do so. And in the *longer term*, the intervention would lead to improved dietary intake and improved nutritional status.

The exact causal pathways will vary depending on the intervention design which should be informed by the context and the particular gender‐related barriers present. While we have focused this systematic review on interventions to improve dietary intake, the logic model includes pathways to improved nutritional status via improved access to essential health services for the prevention of diseases that contribute to malnutrition (e.g., malaria) and the prevention of early pregnancy—a significant contributor to malnutrition among adolescent girls (Black et al., 2013).

Promoting women's empowerment can also potentially lead to adverse effects. For example, adolescent girls, empowered to choose their own diets, may opt to consume low‐nutrient foods, such as sugar‐sweetened beverages (Akseer et al., 2017). There may also be backlash within the household or community in response to adolescent girls’ attempts to assert increased autonomy, which may be perceived as threatening traditional power structures. Capturing the adverse effects of the intervention is a critical component to be explored that will assist in intervention design that maximises benefits and minimises harms.

Underpinning the logic model are the individual‐level, household/community‐level, and macro‐level factors that can moderate the expected outcomes along the causal pathway. At the individual level, socially stratifying factors that contribute to health inequities have the potential to place additional barriers to proper nutrition for adolescent girls and need to be considered in intervention design and evaluation. The PROGRESS‐Plus framework (O'Neill et al., 2014) is a useful pneumonic summarising these factors: Place of residence, Race/ethnicity/culture/language, Occupation, Gender/sex, Religion, Education, Socioeconomic status, and Social capital. The “Plus” stands for other personal characteristics (e.g., age or disability), features of relationships (e.g., children of parents who smoke), and time‐dependent relationships (e.g., recently out of hospital) that can make an individual more vulnerable to poor health. Moving up one level, household and community characteristics can affect intervention design and results as well. Things to consider include household food security and water and sanitation facilities, the availability of health services in the community, women's representation in community leadership or other governing bodies, and other community gender norms, such as son preference. Finally, consideration of the macro‐level context includes such things as the national or regional food environment and food security, national gender‐related laws, policies, and institutional practices such women's rights to land ownership, and women's participation in the economy. The exact strategies necessary to foster agency and opportunity structure are highly context‐specific and point to the importance of understanding not only how women's empowerment affects nutrition outcomes, but also how these contextual factors influence intervention effectiveness.

A final element to consider are the implementation factors regarding the intervention itself (Cargo et al., 2018). These include the intervention setting and delivery platform (e.g., school, health facility, community, etc.), the intervention provider (e.g., teacher, community leader, facility based health worker, community health worker, etc.), how participants were recruited, participant retention strategies and attrition rates, the reach and dose of the intervention, fidelity to intervention design, and the adaptability of the intervention to the local context and circumstances.

### 1.7 Why it is important to do the review

There is a rapidly expanding body of literature on women's empowerment and health, including nutrition. Below we summarise existing reviews of interventions related to women's empowerment and nutrition in low‐ and middle‐income countries. Our systematic review will contribute to the existing evidence‐base by: (a) focusing on the role of empowerment in adolescent girls’ nutrition (an understudied population), (b) attempting to separate out the role of agency‐related and opportunity structure‐related activities in the empowerment process, and (c) systematically reviewing and synthesising the evidence on the contextual and implementation factors that may help to explain the success or failure of empowerment‐based nutrition interventions.

### 1.8 Reviews

van den Bold, Quisumbing, and Gillespie (2015) conducted a review of nutrition‐sensitive structural interventions (cash transfer, agriculture, and microcredit programmes) to assess their impact on women's empowerment and infant and child nutrition, and found that, despite the existence of considerable evidence depicting the associations between women's empowerment and nutritional status, more research is needed to understand the pathways that connect the two concepts. The review did not examine outcomes for adolescent girls.

Similarly, Taukobong et al. (2016) reviewed the literature across six sectors, including nutrition, to assess whether addressing gender inequalities and empowering women and girls improves health and development outcomes. They identified common gender equality and women's empowerment variables that repeatedly emerged as significant predictors of sector outcomes, including control over income, decision‐making power, and education, but also identified the need for more research into mechanisms through which gendered interventions might work. The review did not examine outcomes for adolescent girls.

Brody et al. (2015) conducted a systematic review of economic self‐help group programmes for improving women's empowerment. They concluded that self‐help groups have positive effects on women's economic, social, and political empowerment. Qualitative findings indicated that the poorest women were less likely to participate due to time constraints and fears of racial or caste discrimination. The review did not assess the impact of economic self‐help groups on health or nutrition outcomes for women.

Kumar et al. (2018) conducted a review of women's group‐based programmes and nutritional change in South Asia. They mapped interventions to four conceptual pathways (income, agriculture, health and nutrition behaviour change, and rights) from women's groups to improved nutritional status among women and children. They found the strongest evidence linking the behavior change pathways to improved nutritional status, but also noted a number of challenges in assessing the evidence based including the lack of rigorous studies, difficulties in disentangling the effects of group‐based activities with other aspects of the intervention, a paucity of studies targeting nutritionally vulnerable age groups, and programmes with insufficient reach and duration. They recommend that implementers and evaluators identify a priori which change pathways they anticipate to activate and measure processes and impacts accordingly. The review did not examine outcomes for adolescent girls. conducted a review of women's group‐based programmes and nutritional change in South Asia (Kumar et al., 2018). They mapped interventions to four conceptual pathways (income, agriculture, health and nutrition behaviour change, and rights) from women's groups to improved nutritional status among women and children. They found the strongest evidence linking the behavior change pathways to improved nutritional status, but also noted a number of challenges in assessing the evidence based including the lack of rigorous studies, difficulties in disentangling the effects of group‐based activities with other aspects of the intervention, a paucity of studies targeting nutritionally vulnerable age groups, and programmes with insufficient reach and duration. They recommend that implementers and evaluators identify a priori which change pathways they anticipate to activate and measure processes and impacts accordingly. The review did not examine outcomes for adolescent girls. conducted a review of women's group‐based programmes and nutritional change in South Asia (Kumar et al., 2018). They mapped interventions to four conceptual pathways (income, agriculture, health and nutrition behaviour change, and rights) from women's groups to improved nutritional status among women and children. They found the strongest evidence linking the behavior change pathways to improved nutritional status, but also noted a number of challenges in assessing the evidence based including the lack of rigorous studies, difficulties in disentangling the effects of group‐based activities with other aspects of the intervention, a paucity of studies targeting nutritionally vulnerable age groups, and programmes with insufficient reach and duration. They recommend that implementers and evaluators identify a priori which change pathways they anticipate to activate and measure processes and impacts accordingly. The review did not examine outcomes for adolescent girls. conducted a review of women's group‐based programmes and nutritional change in South Asia (Kumar et al., 2018). They mapped interventions to four conceptual pathways (income, agriculture, health and nutrition behaviour change, and rights) from women's groups to improved nutritional status among women and children. They found the strongest evidence linking the behavior change pathways to improved nutritional status, but also noted a number of challenges in assessing the evidence based including the lack of rigorous studies, difficulties in disentangling the effects of group‐based activities with other aspects of the intervention, a paucity of studies targeting nutritionally vulnerable age groups, and programmes with insufficient reach and duration. They recommend that implementers and evaluators identify a priori which change pathways they anticipate to activate and measure processes and impacts accordingly. The review did not examine outcomes for adolescent girls.

Brandstetter, Rüter, Curbach, and Loss (2015) conducted a systematic review of the various ways of applying the empowerment concept to healthy nutrition in health promotion. The included eight studies that were all based in high‐income countries. They found diversity in the way the concept of empowerment was operationalized, in the integration of other theoretical frameworks (e.g., socio‐cognitive theory), and reporting in the methods of operationalizing and measuring empowerment.

The WHO Guideline, “Implementing effective actions for improving adolescent nutrition” (WHO, 2018a), summarises the global evidence for addressing malnutrition in adolescents. The guideline identifies implementing interventions to empower adolescent girls may prevent early marriage and pregnancy, reduce sexual coercion, and notes that community stakeholders (including teachers and health workers) need to support the empowerment of adolescents to adopt and maintain optimal nutrition and health practices. The guideline further identifies as a research gap the assessment of impact of interventions and policies on autonomy, positive development, empowerment and engagement of adolescents.

Recent systematic reviews of adolescent nutrition interventions (Bhutta et al., 2013; Lassi, Moin, Das, Salam, & Bhutta, 2017; Salam, Hooda, et al., 2016) have shed valuable light on the effectiveness of nutrition interventions in improving adolescent health, but they have not specifically assessed the role of empowerment in adolescent health and nutrition.

### 1.9 Protocols

Vollmer et al. (2017) have published a protocol for a systematic review of the effect of women's economic empowerment on children's health and education.

## 2 OBJECTIVES

The primary objective of the review is to answer the following:

1. Does promoting women's empowerment within nutrition interventions improve the nutritional status of adolescent girls in low‐ and middle‐income countries?
2. What are the factors influencing the success or failure of these interventions?
  The secondary objective is to answer the following:
3. What are the underlying empowerment‐related programme theories that influence intervention design? How is empowerment defined, operationalized, and measured?
4. What is the effect of promoting women's empowerment in nutrition sector interventions on adolescent girls’ empowerment‐related outcomes?
5. What are the potential adverse effects of promoting women's empowerment in nutrition sector interventions on adolescent girls’ health and well‐being?

## 3 METHODOLOGY

This review will apply a segregated mixed methods research synthesis design (MMRS). An MMRS combines qualitative, quantitative, and mix‐method primary‐level studies and applies a mixed methods approach to synthesise and integrate the studies’ results (Heyvaert, Hannes, & Onghena, 2017). This design is more appropriate for the study of interventions that consist of multiple components as it helps to better understand how the different components are related and interact with each other (Heyvaert et al., 2017).

Through a comprehensive literature search, we will identify relevant literature with qualitative and quantitative study designs that will be segregated at the screening phase. Qualitative and quantitative studies will be analysed and synthesised separately, while the implications for practice, policy, and research that will form the discussion and conclusion sections of the review will draw on both the qualitative and quantitative syntheses.

### 3.1 Criteria for including and excluding studies

#### 3.1.1 Types of study designs

The following study designs will be included to answer **Research Question 1** (effectiveness assessment):

- Randomised Controlled Trials (RCTs)
- Cluster Randomised Controlled Trials (cRCT)
- Controlled before and after studies (CBAs)
- (Controlled) interrupted time series (CITS, ITS)
- Propensity score matching (PSM) on baseline covariates
- Regression discontinuity design (RDD)
- Difference in difference using regression techniques (DID)
- Interventions with a synthetic control group
- Other quasi‐experimental designs with at least one comparison group

Studies without an observable comparator or credible means for controlling for selection bias will be excluded.

To answer *Research Question 2*, we will include companion quantitative and qualitative studies that assess the contextual and implementation factors influencing the effectiveness of the studies screened in to the effectiveness assessment (Research Question 1).

We will include qualitative studies that explore the perspectives of intervention participants (adolescent girls), (those who delivered the intervention) providers, or administrators (those who provided oversight or funding to the intervention) on the contextual and implementation factors contributing to intervention success or failure using focus groups, in‐depth interviews or participant observation. Eligible qualitative study designs include case studies, ethnographic research, grounded theory, and other thematic approaches to qualitative data analysis. Qualitative studies that do not report a clear methods and results section will be excluded. This includes opinion pieces and editorials. Eligible quantitative study designs that assess the implementation of included studies are process evaluations, surveys of intervention participants, providers, administrators, and other programme‐related documents such as monitoring and evaluation reports.

Studies that apply a mixed methods design will be included where the qualitative and quantitative study components are reported separately.

#### 3.1.2 Types of participants

The review target population is adolescent girls (10 to 19 years) residing in low‐ and middle‐income countries, regardless of health status.

The classification of countries as low‐ or middle‐income will be based on the World Bank income groups as defined at the time the studies were conducted. Studies undertaken in high‐income countries will be excluded.

#### 3.1.3 Types of interventions

Eligible studies will be empowerment‐based nutrition interventions to improve dietary intake among adolescent girls. More specifically, we will include interventions whose primary aim is to improve dietary intake among adolescent girls by promoting a healthy diet or providing additional micronutrients through fortification or targeted supplementation. Examples of eligible nutrition interventions are:

- Micronutrient supplementation or fortification interventions (iron, folic acid, iron–folic acid (IFA), calcium, vitamin D, vitamin A, zinc, iodine, and multiple micronutrients)
- Nutrition education or counselling interventions, and
- Supplementary nutrition programmes such as school feeding.

To be considered “empowerment‐based,” the nutrition intervention must include one or more activities intended to foster adolescent girls’ agency, and one or more activities to create a support opportunity structure for adolescent girls’ empowerment.

We define activities to foster agency as those that “provide spaces for self‐reflection and identification of important life areas” (Shankar et al., 2019) and equip adolescent girls to make informed strategic life decisions. Examples of activities that may foster adolescent girls’ agency include, but are not limited to:

- Counselling programmes
- Mentorship programmes
- Leadership training
- Life skills training
- Technical or occupational skills training

These programmes may be delivered one‐on‐one (e.g., individual mentorship or counselling) or in a group setting such as girls’ clubs or other peer support or participatory action groups.

We define activities to create a supportive opportunity structure as those that aim to alter the constraining political, economic, socio‐cultural, interpersonal, and/or legal structures (informal or formal) at the household, community, or broader societal levels, as necessary, to support adolescent girls to exercise agency (Alsop & Heinsohn, 2005; Malhotra et al., 2002). Examples of opportunity structure activities include, but are not limited to:

1. *Economic*: Economic activities aim to increase adolescent girls’ access to and control over financial and material resources. These include microcredit programmes, cash transfer programmes, agriculture programmes, homestead or community gardening programmes, and savings and loan programmes.
2. *Socio‐cultural*: Socio‐cultural activities aim to redress discriminatory gender norms, customs and practices that restrict adolescent girls’ ability to exercise agency, most often at the household and community level. Examples include activities to prevent child marriage, programmes to improve freedom of movement, male or in‐law engagement strategies to reduce intra‐household food discrimination, and programmes to support adolescent girls’ completion of secondary education.
3. *Legal*: Legal activities aim to establish laws meant to prevent gender‐based discrimination and protect adolescent girls’ rights. This can include adolescent girls’ rights to education, family planning, employment or inheritance.

Nutrition‐sensitive interventions, such as water, sanitation, and hygiene (WASH) programmes, agriculture programmes including community gardens, cash transfer programmes, food security programmes, and family planning programmes will be considered for inclusion if they serve as a delivery platform for an intervention whose primary aim is to improve dietary intake among adolescent girls by promoting a healthy diet or providing additional micronutrients through fortification or targeted supplementation and include activities to foster adolescent girls’ agency.

#### 3.1.4 Comparison

Studies that have a clearly defined comparison group for evaluation of the treatment effect will be included.

#### 3.1.5 Types of outcome measures

The primary outcomes for the effectiveness assessment (Research Question 1) will be measures of adolescent girls’ nutritional status, including:

- Change in body mass index (BMI)
- Change in BMI‐for‐age z‐score
- Change in mid‐upper arm circumference (MUAC)
- Change in haemoglobin (g/L)
- Change in serum ferritin
- Change in anaemia status
- Change in serum vitamin A

Secondary outcomes measures for Research Question 1 are:

- Changes to dietary intake, including micronutrient intake
- Improved access to a nutritious diet
- Improved health behaviours
- Increased access or use of essential health services

We will only include studies that report at least one outcome for our population of interest.

To answer Research Question 2, our outcomes of interest are the perspectives of intervention participants (adolescent girls), providers (those who delivered the intervention), and administrators (those who provided oversight or funding to the intervention) on the contextual and implementation factors that affected intervention success or failure. These data may be qualitative, in the form of in‐depth interviews, focus groups, or participant observation, or quantitative from surveys, process evaluations, and other project documentation.

To answer Research Question 3, we will extract details on the empowerment‐related theories, as described by primary study authors, that were used to inform the development of the women's empowerment‐related activities. We will extract information on the activities that primary authors describe as promoting women's empowerment and the primary authors’ rationale for adopting specific empowerment‐related activities.

For Research Question 4, we will extract and analyse information on the empowerment dimensions and indicators used in studies to assess the impact of the empowerment‐related activities on adolescent girls’ empowerment outcomes. We will use the following definitions of empowerment dimensions, adapted from Brody et al. (2015) and Malhotra et al. (2002). We will classify empowerment indicators by their respective empowerment dimension for analysis.

- *Economic*: The ability to access, own, and control resources. Potential measures include adolescent girls’ control over own income; relative contribution to family support; access to and control of family resources; participation in paid employment.
- *Political*: The ability to participate politically at the local, regional, or national level. Potential measures include knowledge of the political system and means of access to it; domestic support for political engagement; exercising the right to vote (if of legal age).
- *Socio‐cultural*: The ability to overcome discriminatory gender norms at the household and community levels. Potential measures include: Adolescent girls’ freedom of movement; lack of discrimination against daughters in the household; household commitment to educating daughters.
- *Intrafamilial*: The ability to exert power and influence in the household. Potential measures include control over sexual relations; ability to make childbearing decisions, use contraception, access abortion; control over spouse selection and marriage timing; freedom from domestic violence.
- *Legal*: The ability to access rights and entitlements under the law. Potential measures include knowledge of legal rights; domestic support for exercising rights.
- *Psychological*: The ability to make choices and act on them. Potential measures include self‐esteem; self‐ efficacy; psychological well‐being.

Finally, to answer Research Question 5, we will extract information on the negative or adverse effects of promoting women's empowerment in nutrition interventions for adolescent girls. These include gender‐based violence, discrimination, demotivation, and adoption of unhealthy eating habits (e.g., increased consumption of sugar‐sweetened beverages).

#### 3.1.6 Duration of follow‐up

We will include studies of any follow‐up duration and will conduct sensitivity analyses by length of follow‐up to test the sustainability of treatment effect.

#### 3.1.7 Types of settings

Interventions delivered at home, in the community, in school, in the workplace, or in health facilities will be eligible for inclusion.

#### 3.1.8 Language

No language restrictions will be applied.

#### 3.1.9 Publication date

No publication data restrictions will be applied.

### 3.2 Search strategy

A comprehensive search strategy was developed with the assistance of an information specialist (Appendix B). Keywords used to develop the search strategy include variations on the following: “power,” “empowerment,” “self‐efficacy,” “self‐determination,” “personal autonomy,” “agency,” “women's status,” “women's rights,” “malnutrition,” “underweight,” “overweight,” “diet,” “micronutrient,” “vitamin,” “nutrition education,” “school feeding,” “food supplementation,” “women,” “girls,” “female,” “maternal,” “adolescent,” “teenager,” “youth.”

#### 3.2.1 Databases

An experienced medical information specialist will develop and test the search strategy using an iterative process in consultation with the review authors. Another senior information specialist will peer review the search strategy prior to execution using the PRESS Checklist.

We will use a combination of controlled vocabulary (e.g., “Power [Psychology],” “Women's Rights,” “Nutritional Status”) and key words (e.g., empower, female status, and diet) for the concepts in all searches. We will apply the Cochrane filter for low‐ and middle‐income countries. Vocabulary and syntax will be adjusted as necessary across the databases. We will remove animal‐only and opinion pieces from the results whenever possible.

Using the OVID platform, we will search Ovid MEDLINE®, including Epub Ahead of Print and In‐Process & Other Non‐Indexed Citations, Embase Classic + Embase, PsycINFO, and the following EBM Reviews databases: Cochrane Central Register of Controlled Trials, Cochrane Database of Systematic Reviews, Database of Abstracts of Reviews of Effects, Health Technology Assessment, and the NHS Economic Evaluation Database. We will also search CINAHL (Ebsco platform), Web of Science, and Popline.

We will document the search process in enough detail to ensure that it can be reported correctly in the review/update, including reporting the month and year the search began and ended.

##### Grey literature and hand searching:

To identify potentially relevant unpublished materials, we will contact the following research groups and organisations, and/or consult their respective websites:

- World Health Organisation Library (includes LILACS)
- Epistemonikos
- 3ie Impact and Systematic Review repositories
- E‐Library of Evidence for Nutrition Actions (eLENA)
- UNICEF
- World Food Programme
- UNFPA
- International Food Policy Research Institute (IFPRI)
- Global Alliance for Improved Nutrition (GAIN)
- Nutrition International
- SPRING Project
- International Centre for Research on Women (ICRW)
- UN Women
- Gender and Adolescence: Global Evidence (GAGE) Programme
- U.K. Department of International Development (DfID)
- Bill and Melinda Gates Foundation
- Plan International
- CARE
- Save the Children
- World Vision
- Young Lives
- Emergency Nutrition Network

##### Citation and reference lists

The citation and reference lists of included references, including other reviews, will be searched. “Related articles” features of searched databases will be used, where applicable. We will conduct forward citation tracking using Scopus.

##### Contacting experts

We will contact authors of included studies to ask for suggested studies.

##### Screening of studies

Study selection will be conducted in duplicate by two independent reviewers using the Covidence platform (www.covidence.org). Titles and abstracts resulting from the search strategy will be independently screened by two reviewers in the first phase, followed by independent full‐text review of eligible studies, also in duplicate. Any discrepancies between the independent reviewers will be resolved by consensus, and in cases of disagreement, a third author will be consulted. A PRISMA study selection flow chart (Moher, Liberati, Tetzlaff, & Altman, 2009) will be prepared, and a list of excluded studies will be compiled detailing the reason for each study's exclusion.

To minimise the risk of excluding eligible studies, we will screen for nutrition‐related activities only at the title and abstract phase. In other words, we screen in all studies that aim to improve dietary intake among adolescent girls by promoting a healthy diet or providing additional micronutrients through fortification or targeted supplementation when screening titles and abstracts. At the full‐text screening stage we will further screen for the agency and opportunity structure components to determine study eligibility.

In instances where articles do not provide a sufficient description of the intervention to determine its eligibility, we will look for companion articles describing the intervention or contact the authors for additional information. Where we cannot obtain additional information on the intervention, the studies will be excluded from the review.

### 3.3 Description of methods used in primary research

The following study is an example of the expected eligible primary research methods:

Hewett et al. (2017): Multiarm cluster‐RCT across 10 sites in four provinces of Zambia with randomisation to four different study arms (Intervention Arm 1, Intervention Arm 2, Intervention Arm 3, Control). The sites were evenly split between urban and rural settings (five sites each). The intervention assessed the effectiveness of the Adolescent Girls Empowerment Programme (AGEP), which consisted of weekly mentor‐led girls’ group meetings of 20 to 30 adolescent girls, participating over 2 years using curricula on sexual and reproductive health and life skills, financial literacy, and nutrition. Two additional components were a health voucher and bank account. Clusters were defined by national Census Supervisory Areas (CSAs) that were randomly selected to the experimental and control arms through a public lottery. Allocation concealment was unclear. Baseline behavioural surveys and biological specimen collection were conducted at the beginning of the trial, reassessed immediately after the programme ended (2015), and evaluated again at the 2‐year follow‐up point (2017). It is unclear if a cluster‐adjustment method was applied (Hewett et al., 2017).

### 3.4 Criteria for determination of independent findings

Where studies report different outcomes, these will be pooled in separate meta‐analyses. If there are several publications reporting on the same study, we will use effect sizes from the most recent publication. In cases where several studies use the same data set or multiple outcomes are reported within the same study, we will select the study that provides the lowest risk of bias in attributing impact. Where studies include multiple outcome measures to assess related outcome constructs, we will select the outcome that appears to most accurately reflect the outcome construct of interest (Macdonald, Higgins, & Ramchandani, 2006). For studies in which multiple effects over time are reported, a variance estimation meta‐analysis will be conducted. For studies having multiple treatments with only one control group, where the treatments might represent separate treatment constructs, we will calculate the effect size for each pair of treatment versus control separately.

### 3.5 Details of study coding categories

The quality assessment of included studies and data extraction will be done by two independent reviewers.

#### 3.5.1 Quality assessment

We will use the risk of bias tool developed by the International Development Coordinating Group (IDCG) secretariat to assess risk of bias, similar to Baird, Ferreira, Özler, and Woolcock (2013). This tool has been developed to assess the risk of bias for a range of experimental and quasi‐experimental studies. The tool assesses risk of bias in the following categories:

- Selection bias and confounding
- Spill‐overs/crossovers/contamination
- Outcome reporting
- Analysis reporting
- Other risks of bias, including unit of analysis errors, detection bias and placebo effects, motivation and courtesy biases, coherence of results, and others.

Judgements made on risk of bias in quantitative studies will be supported by specific information extracted from the study being assessed. An overall level of evidence quality (high, moderate, low, and very low) for the entire body of evidence will be assigned as part of the GRADE process (Atkins et al., 2004).

We will assess the quality of qualitative studies using the Critical Appraisal Skills Programme (CASP) qualitative appraisal research tool (Critical Appraisal Skills Programme, 2013). The GRADE‐CERQual approach will be used to assess the overall confidence in the qualitative evidence synthesis (Lewin et al., 2015). GRADE‐CERQual provides an assessment of confidence regarding the extent to which the research finding is likely to be substantially different from the phenomenon of interest. A level of confidence in the review findings will be assigned, ranging from high, moderate, low to very low confidence (Lewin et al., 2015).

#### 3.5.2 Data extraction

Data extraction will be conducted in duplicate by two independent reviewers. Both reviewers will use a prepiloted data extraction form. Discrepancies between the two extractors will be resolved through discussion or by consultation with a third reviewer. See Appendix C for draft codebooks that will guide data extraction.

The following information on intervention design will be extracted:

- The intervention setting, e.g., school, community, home, workplace so forth.
- The intervention administrator, e.g., foreign government, national or local government, nongovernmental organisation, community‐based organisation so forth.
- The intervention provider, e.g., community health worker, health facility staff, teachers, peers so forth.
- Descriptions of any training given to intervention providers before and during the intervention
- Description of any prior needs assessment to inform intervention design
- Participant recruitment procedures
- Participant attrition rate and reasons for attrition
- Activities undertaken to design the intervention in a culturally‐sensitive manner
- Intervention reach (the degree to which participants are present and participate)
- Intervention dose (frequency, intensity and duration of intervention delivery to participants)
- Intervention integrity/fidelity (degree to which the intervention was delivered according to original design)
- Intervention adaptation (adaptation during implementation to respond to changing circumstances)
- Contamination (unintentional delivery of intervention to comparison group or failure to provide intervention to intervention group
- Cointervention (unintentional delivery of another intervention to study population)
- Participant engagement (active participation and receptivity to the intervention)
- Intervention quality
- Contextual factors that shape implementation effectiveness (e.g., level of food insecurity)
- Authors’ definition of empowerment and rationale for incorporating empowerment‐related activities

For quantitative outcomes, we will extract the following:

- For dichotomous outcomes, we will extract the total number of participants in the treatment group and the total number experiencing the event to allow the calculation of odds ratios and relative risks (or data necessary for their calculation).
- For normally‐distributed, continuous outcomes, we will extract means, standard deviations (or data necessary for their estimation) and the number of participants in each treatment group.
- For skewed continuous data, we will extract medians, ranges, and *p* values. Outcomes that were measured at different time points will be recorded separately.

For measures of empowerment, we will extract definitions of the measures used, the empowerment dimensions being measured (according to the primary authors), and the primary authors’ rationale for outcome selection.

We will extract data on socioeconomic status, education level, race/ethnicity/caste, place of residence (urban, rural, slum, remote), and other potential effect moderators for subgroup analyses based on the PROGRESS‐Plus framework (O'Neill et al., 2014).

Quantitative data will be entered into RevMan5 and checked for accuracy.

For qualitative studies, we will extract the views, experiences, and opinions of intervention participants, implementers and administrators on factors influencing the success or failure of interventions. Emphasis will be placed on ascertaining the feasibility, appropriateness, and meaningfulness of the women's empowerment components of the intervention.

### 3.6 Statistical procedures and conventions

Quantitative data will be synthesised using meta‐analysis, where appropriate. We expect a high level of heterogeneity due to the fact that studies may employ a variety of different nutrition, agency, and opportunity structure activities. Consequently, we will use a random‐effects model to produce an overall summary estimate, if an average treatment effect across studies is considered meaningful. Where meta‐analysis is not possible or is deemed inappropriate, results will be reported using narrative synthesis, giving effect sizes and confidence intervals, where applicable (Popay et al., 2006).

We will assess heterogeneity among studies by first examining the heterogeneity at face‐value in terms of the studies’ populations, interventions, and outcomes. Second, we will use *τ* ^2^ to statistically test heterogeneity between studies. *τ* ^2^ is the variance of the effect size parameters across the population of studies and reflects the variance of the true effect sizes (Borenstein, Hedges, Higgins, & Higgins, 2009).

The accuracy of numeric data will be checked by comparing the magnitude and direction of effects reported by studies and how they are presented in the review. A statistically nonsignificant *p* value will be interpreted as a finding of uncertainty unless confidence intervals are sufficiently narrow to rule out an important magnitude of effect.

Our base‐case analysis will include all interventions regardless of nutrition, agency or opportunity structure intervention. If there is a sufficient number of studies, we will conduct the following sensitivity analyses based on intervention design (Table 2).

**Table 2:** Sensitivity tests by intervention design

Test	Nutrition	Agency	Opportunity structure
Base case	All nutrition activities combined	All agency activities combined	All opportunity structure activities combined
Test 1	All nutrition activities combined	All agency activities combined	Opportunity structure activities split by dimension
Test 2	All nutrition activities combined	Agency activities split by group vs nongroup activities	All opportunity structure activities combined
Test 3	Nutrition activities split by promotion of a healthy diet, providing additional micronutrients, or a combination of both	All agency activities combined	All opportunity structure activities combined

Should the number of studies warrant, we will further split the interventions and run further sensitivity analyses. For example, we will consider sensitivity tests that break down all three intervention elements. Otherwise, we will provide a narrative summary.

We will combine experimental and quasi‐experimental designs for analysis and conduct a sensitivity analysis by study design. We will use David Wilson's effect size calculator for quasi‐experimental study outcomes to allow for combining of experimental and quasi‐experimental study outcomes for meta‐analysis (Lipsey, 2001). Primary and secondary outcome data will be extracted and analyzed separately. Binary outcomes (e.g., anaemia status) will be analyzed using risk ratios (±95% confidence interval [CI]). Continuous outcomes (e.g., height and weight) will be analyzed using mean differences (±95% CI) and standardised mean differences when different units are used (e.g., measures of dietary diversity or empowerment indicators). In the random effects meta‐analysis, Mantel‐Haenszel (M‐H) methods will be used for binary outcomes, and the Inverse‐Variance (I‐V) method will be used for continuous outcomes. Where studies use different metrics for the same outcome, e.g., anemia status (binary) vs haemoglobin (continuous), we will convert to the same metric using Borenstein's conversion formulae (Borenstein et al., 2009) and synthesise.

Unit of analysis errors will be investigated to ensure estimates are properly adjusted for clustering. Where analyses are not adjusted for clustering, estimates will be adjusted using values of intra‐cluster correlations from the literature. An intention‐to‐treat analysis will be conducted. We will document how authors treated missing data, and the effect of missing data on the overall results will be assessed through sensitivity analysis.

We will conduct moderator analyses on the following:

- Risk of bias (low, unclear, and high)
- Study duration
- Low‐income country vs. middle‐income country
- Geographic region (Africa, Asia, and Latin America)
- Study design (RCT vs. NRS)
- Group vs. nongroup interventions
- Marital status (single, currently partnered, divorcee/separated/widowed)
- Number of children (0 vs. 1+)
- Education level (none, some primary, primary complete, some secondary, secondary complete, and higher)
- Age (10–14 y vs. 15–19 y)

A formal statistical test will be used to test differences between outs. For subgroups defined by binary or nominal categories, we will use the Cochran *Q*‐test. For ordinal categories, multi‐level meta‐analysis will be conducted. The results of all subgroup testing will be reported, regardless of results.

If more than ten studies meet our eligibility criteria, we will assess the presentation of publication bias using a visual inspection of funnel plots. Statistical support will be provided by a statistician, and meta‐analyses will be conducted using RevMan5 software (The Cochrane Collaboration). Results will be displayed using forest plots.

The level of evidence will be considered when formulating the review's conclusions. Where possible, differences in results will be explained by giving a description of likely explanatory factors. We will prepare a GRADE summary of findings table (Schunemann, Brozek, Guyatt, & Oxman, 2013).

### 3.7 Treatment of qualitative research

We will use the “best fit” framework synthesis method to synthesise data (Carroll, Booth, Leaviss, & Rick, 2013; Harden et al., 2018). The “best fit” framework synthesis method allows for the testing or adaption of an existing model to a potentially different population. For this review, we have adapted existing models for nutrition and women's empowerment to develop a logic model (described earlier) for adolescent girls’ empowerment and nutrition, and plan to test its applicability through the systematic review process. We will code qualitative data from the review's included studies against the conceptual framework at each level of the conceptual framework:

1. Elements of the approach
  a. Nutrition‐related activities
  b. Agency‐related activities
  c. Opportunity structure activities
2. Outputs (or implementation factors)
  a. Intervention setting
  b. Intervention administrator
  c. Intervention provider
  d. Provider training
  e. Prior needs assessment to inform intervention design
  f. Participant recruitment procedures
  g. Participant attrition rate and reasons for attrition
  h. Activities undertaken to design the intervention in a culturally‐sensitive manner
  i. Intervention reach
  j. Intervention dose
  k. Intervention integrity/fidelity
  l. Intervention adaptation
  m. Contamination
  n. Cointervention
  o. Participant engagement
  p. Intervention quality
3. Shorter‐term outcomes
  a. Increased ability and motivation to make and act on informed choices to improve nutritional status
  b. Supportive home and community environment to support adolescent girls’ choices
  c. Improved awareness, knowledge and skills for the adoption of a healthy diet and positive health behaviours
4. Intermediate‐term outcomes
  a. Increased decision‐making power
  b. Increased access to or control over resources
  c. Protection and fulfilment of human rights
  d. Improved access to a nutritious diet
  e. Increase use of essential health services
  f. Improved health behaviours
5. Longer‐term outcomes
  a. Improved dietary intake
  b. Reduced risk of disease, infection, injury, and early pregnancy
  c. *Adverse effects*
6. Contextual factors
  a. Macro‐level
  b. Community level
  c. Household level
  d. Individual level

Inductive, thematic analysis techniques will be used to synthesise data that do not align to the existing themes in the logic model. Emphasis will be placed on understanding the role that promoting women's empowerment in the nutrition sector programme had on intervention success or failure, with a focus on aspects regarding the feasibility, appropriateness and meaningfulness of the empowerment‐related activities or strategies that were employed.

The conclusions drawn from the quantitative and qualitative syntheses will be combined to inform the review's final discussion and conclusions. The logic model will be revised based on the review's conclusions. The review's discussion will include reflections on the review's policy and future research implications.

## ROLES AND RESPONSIBILITIES

Please give a brief description of content and methodological expertise within the review team. It is recommended to have at least one person on the review team who has content expertise, at least one person who has methodological expertise and at least one person who has statistical expertise. It is also recommended to have one person with information retrieval expertise. Please note that this is the *recommended optimal* review team composition.

- Content: A. Y. R., Z. A. B., C. V., E. K., A. K. R., C. M. K.
- Systematic review methods: G. A. W., V. W., E. K.
- Statistical analysis: G. A. W., M. T.
- Information retrieval: B. S.

## SOURCES OF SUPPORT

The lead author (A. Y. R.) is the recipient of a graduate studentship award from the Bruyère Research Institute, and a PhD grant from Nutrition International.

## DECLARATIONS OF INTEREST

A. Y. R., C. M. K., A. K. R., Z. B., C. V., E. B., C. K., and L. S. have no conflicts of interest to declare. V. W. is the Editor‐in‐Chief of the Campbell Collaboration.

## PRELIMINARY TIMEFRAME

Approximate date for submission of the systematic review: March 2020.

## PLANS FOR UPDATING THE REVIEW

The review will be updated every two years. AYR will be responsible for updating the review.

## AUTHOR DECLARATION

### Authors’ responsibilities

By completing this form, you accept responsibility for preparing, maintaining and updating the review in accordance with Campbell Collaboration policy. Campbell will provide as much support as possible to assist with the preparation of the review.

A draft review must be submitted to the relevant Coordinating Group within two years of protocol publication. If drafts are not submitted before the agreed deadlines, or if we are unable to contact you for an extended period, the relevant Coordinating Group has the right to deregister the title or transfer the title to alternative authors. The Coordinating Group also has the right to deregister or transfer the title if it does not meet the standards of the Coordinating Group and/or Campbell.

You accept responsibility for maintaining the review in light of new evidence, comments and criticisms, and other developments, and updating the review at least once every 5 years, or, if requested, transferring responsibility for maintaining the review to others as agreed with the Coordinating Group.

### Publication in the Campbell Library

The support of the Coordinating Group in preparing your review is conditional upon your agreement to publish the protocol, finished review, and subsequent updates in the Campbell Library. Campbell places no restrictions on publication of the findings of a Campbell systematic review in a more abbreviated form as a journal article either before or after the publication of the monograph version in Campbell Systematic Reviews. Some journals, however, have restrictions that preclude publication of findings that have been, or will be, reported elsewhere and authors considering publication in such a journal should be aware of possible conflict with publication of the monograph version in Campbell Systematic Reviews. Publication in a journal after publication or in press status in Campbell Systematic Reviews should acknowledge the Campbell version and include a citation to it. Note that systematic reviews published in Campbell Systematic Reviews and co‐registered with Cochrane may have additional requirements or restrictions for co‐publication. Review authors accept responsibility for meeting any co‐publication requirements.

## APPENDIX B MEDLINE SEARCH STRATEGY

1 “ Power (Psychology)”/ (158608)
2 Attitude/ (107645)
3 Attitude to Health/ (190744)
4 ((attitud* or opinion*) adj3 (diet* or eating or feeding or food? or health* or malnutrit* or malnourish* or meal? or nutrient? or nutrition or nutritive or snack?)).tw,kf. (41819)
5 Intention/ (171233)
6 ((intention? or intent? or intend*) adj3 (diet* or eating or feeding or food? or health* or malnutrit* or malnourish* or meal? or nutrient? or nutrition or nutritive or snack?)).tw,kf. (8807)
7 Motivation/ (216063)
8 (motivat* adj3 (diet* or eating or feeding or food? or health* or malnutrit* or malnourish* or meal? or nutrient? or nutrition or nutritive or snack?)).tw,kf. (13802)
9 (encourag* adj3 (diet* or eating or feeding or food? or health* or malnutrit* or malnourish* or meal? or nutrient? or nutrition or nutritive or snack?)).tw,kf. (14391)
10 Self Efficacy/ (92885)
11 Personal Autonomy/ (29331)
12 Self Concept/ (184924)
13 (capable or capabilit* or emancipat* or empower* or agency or self‐efficac* or power* or autonomy or (self adj2 determin*) or (self adj2 concept*) or (self adj2 confiden*) or (self adj2 percept*) or (self adj2 esteem)).tw,kf. (2505725)
14 (abilit* adj3 (act or acted or acting or acts or action?)).tw,kf. (7288)
15 Women's Rights/ (15820)
16 ((wom#n* adj2 right?) or (wom#n* adj2 status*) or (girl* adj2 right?) or (girl* adj2 status*) or (female? adj2 right?) or (female? adj2 status*) or (wife adj2 right?) or (wife adj2 status*) or (mother* adj2 right?) or (mother* adj2 status*) or (wives adj2 right?) or (wives adj2 status*)).tw,kf. (36459)
17 ((wom#n* or girl* or female? or mother* or wife or wives) adj2 (engag* or involv* or participat*)).tw,kf. (74261)
18 (active$2 adj2 (engag* or involv* or participat*)).tw,kf. (61724)
19 participatory.tw,kf. (35824)
20 ((wom#n* or girl* or female? or mother* or wife or wives) adj3 (advoca* or input* or ombuds* or represent*)).tw,kf. (21145)
21 ((citizen* or communit* or gender* or public or village?) adj3 (advoca* or input* or ombuds* or represent*)).tw,kf. (31601)
22 Gender Identity/ (41227)
23 ((wom#n* or girl* or female? or mother* or wife or wives) adj3 role?).tw,kf. (28904)
24 Social Norms/ (10338)
25 ((social* or societ* or cultural* or group?) adj3 (custom* or norm?)).tw,kf. (36015)
26 or/1–25 (3571314)
27 Nutritional Requirements/ (37442)
28 Nutritional Status/ (105385)
29 Nutritive Value/ (30809)
30 Adolescent Nutritional Physiological Phenomena/ (1838)
31 Maternal Nutritional Physiological Phenomena/ (13721)
32 malnutrition/ (75398)
33 exp Deficiency Diseases/ (260675)
34 (avitaminos#s or hypovitaminos#s or hypo‐vitaminos#s).tw,kf. (8831)
35 ((ascorbic acid? or ferrous ascorbate or hybrin or magnesium ascorbate or magnesium ascorbicum or magnesium di‐L‐ascorbate or magnorbin or sodium ascorbate) adj3 (deficien* or deficit? or inadequa* or insufficien* or intak* or lack* or supplement*)).tw,kf. (4094)
36 scurvy.tw,kf. (4345)
37 ((all‐trans‐retinol or aquasol a or retinol) adj3 (deficien* or deficit? or inadequa* or insufficien* or intak* or lack* or supplement*)).tw,kf. (1959)
38 ((bursine or choline or fagine or vagine) adj3 (deficien* or deficit? or inadequa* or insufficien* or intak* or lack* or supplement*)).tw,kf. (7851)
39 ((folacin or folate or folic acid? or folvite or IFA or pteroylglutamic acid?) adj3 (deficien* or deficit? or inadequa* or insufficien* or intak* or lack*or supplement*)).tw,kf. (17888)
40 (hyperhomocystein?emi* or hyper‐homocystein?emi*).tw,kf. (15262)
41 ((niacin? or enduracin or induracin or lithium nicotinate or nicamin or nico‐400 or nicobid or nicocap or nicolar or nicotinate or nicotinic acid? or wampocap) adj3 (deficien* or deficit? or inadequa* or insufficien* or intak* or lack* or supplement*)).tw,kf. (1819)
42 ((tryptophan or ardeydorm or ardeytropin or l‐tryptophan or levotryptophan or lyphan or naturruhe or optimax or pms‐tryptophan or trofan or tryptacin or tryptan or ratio‐tryptophan) adj3 (deficien* or deficit? or inadequa* or insufficien* or intak* or lack* or supplement*)).tw,kf. (2802)
43 pellagra.tw,kf. (2774)
44 (riboflavin? adj3 (deficien* or deficit? or inadequa* or insufficien* or intak* or lack* or supplement*)).tw,kf. (3255)
45 ((thiamine? or aneurin or thiamin) adj3 (deficien* or deficit? or inadequa* or insufficien* or intak* or lack* or supplement*)).tw,kf. (7920)
46 (pyridoxine? adj3 (deficien* or deficit? or inadequa* or insufficien* or intak* or lack* or supplement*)).tw,kf. (2773)
47 overnutrition/ (5853)
48 exp Obesity/ (711110)
49 growth Disorders/ (26038)
50 ((growth adj2 stunt$2) or stunting).tw,kf. (14027)
51 (nutrient? or nutrition* or nutritive or nourish* or malnutrit* or malnourish* or undernutrition or undernourish* or overnutrition or overnourish* or obese or obesity or wasting or underweight or under weight or overweight or over weight).tw,kf. (1769168)
52 anemia/ (242868)
53 exp Anemia, Hypochromic/ (46742)
54 (an?emic or an?emia* or chloros#s).tw,kf. (387455)
55 Feeding Behavior/ (172106)
56 ((eating or feeding or food or meal? or snack*) adj3 (behavio?r* or habit? or habitual* or pattern* or practice?)).tw,kf. (141785)
57 diet/ (402699)
58 (diet or dieted or diets or dietary or dieting).tw,kf. (1174789)
59 energy Intake/ (93531)
60 ((calori* or energy or protein?) adj3 intak*).tw,kf. (115306)
61 healthy Diet/ (4701)
62 ((eating or feeding or food? or meal? or snack*) adj3 (healthy or unhealthy)).tw,kf. (39237)
63 exp Micronutrients/ (688381)
64 (micronutri* or micro‐nutri*).tw,kf. (35383)
65 (trace element? or trace mineral? or micromineral? or micro‐mineral?).tw,kf. (44266)
66 Calcium, Dietary/ (31557)
67 Chromium/ (59565)
68 Copper/ (178475)
69 Iodine/ (75382)
70 Iron/ (259984)
71 Manganese/ (74289)
72 Molybdenum/ (21257)
73 Selenium/ (58025)
74 Zinc/ (170532)
75 (calcium or chromium or copper or fluoride or iodine or iron or manganese or molybdenum or selenium or zinc).tw,kf. (1924054)
76 vitamin*.tw,kf. (512331)
77 exp Dietary Supplements/ (89126)
78 ((eating or feeding or food? or meal? or snack*) adj3 supplement*).tw,kf. (25906)
79 n?utr#ceutical?.tw,kf. (14005)
80 Food, Fortified/ (11093)
81 ((fortif* or enrich* or functional) adj2 (eating or feeding or food? or meal? or snack*)).tw,kf. (25026)
82 ((home? or school* or work*) adj2 (ate or eaten or eating or eats or breakfast* or lunch* or dinner* or supper* or feed* or food? or meal?)).tw,kf. (19850)
83 Food Supply/ (28920)
84 ((eating or feeding or food? or meal? or snack*) adj3 (secure* or securit* or insecure* or insecurit* or supply* or supplie*)).tw,kf. (31860)
85 or/27–84 (6032125)
86 26 and 85 (298093)
87 Developing Countries/ (159704)
88 Africa/ or Asia/ or Caribbean/ or West Indies/ or South America/ or Latin America/ or Central America/ (228463)
89 (Afghanistan or Albania or Algeria or Angola or Argentina or Armenia or Armenian or Azerbaijan or Bangladesh or Benin or Byelarus or Byelorussian or Belarus or Belorussian or Belorussia or Belize or Bhutan or Bolivia or Bosnia or Herzegovina or Hercegovina or Botswana or Brazil or Bulgaria or Burkina Faso or Burkina Fasso or Upper Volta or Burundi or Urundi or Cambodia or Khmer Republic or Kampuchea or Cameroon or Cameroons or Cameron or Camerons or Cape Verde or Central African Republic or Chad or China or Colombia or Comoros or Comoro Islands or Comores or Mayotte or Congo or Zaire or Costa Rica or Cote d’Ivoire or Ivory Coast or Cuba or Djibouti or French Somaliland or Dominica or Dominican Republic or East Timor or East Timur or Timor Leste or Ecuador or Egypt or United Arab Republic or El Salvador or Eritrea or Ethiopia or Fiji or Gabon or Gabonese Republic or Gambia or Gaza or Georgia Republic or Georgian Republic or Ghana or Grenada or Guatemala or Guinea or Guiana or Guyana or Haiti or Honduras or India or Maldives or Indonesia or Iran or Iraq or Jamaica or Jordan or Kazakhstan or Kazakh or Kenya or Kiribati or Korea or Kosovo or Kyrgyzstan or Kirghizia or Kyrgyz Republic or Kirghiz or Kirgizstan or Lao PDR or Laos or Lebanon or Lesotho or Basutoland or Liberia or Libya or Macedonia or Madagascar or Malagasy Republic or Malaysia or Malaya or Malay or Sabah or Sarawak or Malawi or Mali or Marshall Islands or Mauritania or Mauritius or Agalega Islands or Mexico or Micronesia or Middle East or Moldova or Moldovia or Moldovian or Mongolia or Montenegro or Morocco or Ifni or Mozambique or Myanmar or Myanma or Burma or Namibia or Nepal or Netherlands Antilles or Nicaragua or Niger or Nigeria or Muscat or Pakistan or Palau or Palestine or Panama or Paraguay or Peru or Philippines or Philipines or Phillipines or Phillippines or Papua New Guinea or Romania or Rumania or Roumania or Rwanda or Ruanda or Saint Lucia or St Lucia or Saint Vincent or St Vincent or Grenadines or Samoa or Samoan Islands or Navigator Island or Navigator Islands or Sao Tome or Senegal or Serbia or Montenegro or Seychelles or Sierra Leone or Sri Lanka or Solomon Islands or Somalia or Sudan or Suriname or Surinam or Swaziland or South Africa or Syria or Tajikistan or Tadzhikistan or Tadjikistan or Tadzhik or Tanzania or Thailand or Togo or Togolese Republic or Tonga or Tunisia or Turkey or Turkmenistan or Turkmen or Uganda or Ukraine or Uzbekistan or Uzbek or Vanuatu or New Hebrides or Venezuela or Vietnam or Viet Nam or West Bank or Yemen or Zambia or Zimbabwe).tw,kf. (2468939)
90 exp africa/ or exp africa, northern/ or algeria/ or egypt/ or libya/ or morocco/ or tunisia/ or exp “africa south of the sahara”/ or africa, central/ or cameroon/ or central african republic/ or chad/ or congo/ or “democratic republic of the congo”/ or equatorial guinea/ or gabon/ or africa, eastern/ or burundi/ or djibouti/ or eritrea/ or ethiopia/ or kenya/ or rwanda/ or somalia/ or south sudan/ or sudan/ or tanzania/ or uganda/ or africa, southern/ or angola/ or botswana/ or lesotho/ or malawi/ or mozambique/ or namibia/ or south africa/ or swaziland/ or zambia/ or zimbabwe/ or africa, western/ or benin/ or burkina faso/ or cape verde/ or cote d'ivoire/ or gambia/ or ghana/ or guinea/ or guinea‐bissau/ or liberia/ or mali/ or mauritania/ or niger/ or nigeria/ or senegal/ or sierra leone/ or togo/ or americas/ or exp caribbean region/ or exp west indies/ or exp central america/ or belize/ or costa rica/ or el salvador/ or guatemala/ or honduras/ or nicaragua/ or panama/ or panama canal zone/ or latin america/ or mexico/ or exp south america/ or argentina/ or bolivia/ or brazil/ or chile/ or colombia/ or ecuador/ or french guiana/ or guyana/ or paraguay/ or peru/ or suriname/ or uruguay/ or venezuela/ or asia/ or asia, central/ or kazakhstan/ or kyrgyzstan/ or tajikistan/ or turkmenistan/ or uzbekistan/ or exp asia, southeastern/ or borneo/ or brunei/ or cambodia/ or timor‐leste/ or indonesia/ or laos/ or malaysia/ or mekong valley/ or myanmar/ or philippines/ or singapore/ or thailand/ or vietnam/ or asia, western/ or bangladesh/ or bhutan/ or india/ or sikkim/ or middle east/ or afghanistan/ or bahrain/ or iran/ or iraq/ or israel/ or jordan/ or kuwait/ or lebanon/ or oman/ or qatar/ or saudi arabia/ or syria/ or turkey/ or united arab emirates/ or yemen/ or nepal/ or pakistan/ or sri lanka/ or far east/ or china/ or beijing/ or macau/ or tibet/ or korea/ or mongolia/ or taiwan/ or indian ocean islands/ or comoros/ or madagascar/ or mauritius/ or reunion/ or seychelles/ or pacific islands/ or exp melanesia/ or exp micronesia/ or polynesia/ or pitcairn island/ or exp samoa/ or tonga/ or prince edward island/ or west indies/ or “antigua and barbuda”/ or bahamas/ or barbados/ or cuba/ or dominica/ or dominican republic/ or grenada/ or guadeloupe/ or haiti/ or jamaica/ or martinique/ or netherlands antilles/ or puerto rico/ or “saint kitts and nevis”/ or saint lucia/ or “saint vincent and the grenadines”/ or “trinidad and tobago”/ or united states virgin islands/ or oceania/ (2424512)
91 ((developing or less* developed or under developed or underdeveloped or middle income or low* income or underserved or under served or deprived or poor*) adj (countr* or nation? or population? or region? or world or state*)).tw,kf. (262888)
92 ((developing or less* developed or under developed or underdeveloped or middle income or low* income or poor*) adj econom*).tw,kf. (3030)
93 (low* adj (gdp or gnp or gross domestic or gross national)).tw,kf. (641)
94 (low adj3 middle adj3 countr*).tw,kf. (31752)
95 (lmic or lmics or third world or lami countr*).tw,kf. (16026)
96 transitional countr*.tw,kf. (457)
97 or/87–96 (3718691)
98 86 and 97 (32745)
99 Women/ (8405706)
100 Pregnant Women/ (69649)
101 Female/ and (Adolescent/ or Adult/ or Young Adult/) (9600899)
102 (woman* or women* or matern* or mother* or wife or wives or pregnan*).tw,kf. (4328988)
103 ((female? or girl*) adj5 (adolescen* or adult* or child‐bearing or childbearing or highschool* or school* or teen* or marry* or marriage* or marrie? or menstru* or post‐pubescen* or postpubescen* or post‐puberty or postpuberty or reproductive or spous* or youth* or young adult?)).tw,kf. (286629)
104 ((female? or girl or girls) adj2 (ten year? or ten yr? or 10 year? or 10 yr? or eleven year? or eleven yr? or 11 year? or 11 yr? or twelve year? or twelve yr? or 12 year? or 12 yr?)).tw,kf. (28995)
105 ((female? or girl or girls) adj2 (age? or old) adj3 (“10” or ten or “11” or eleven or “12” or twelve)).tw,kf. (66012)
106 young girl?.tw,kf. (10028)
107 or/99–106 (15081858)
108 98 and 107 (19470)
109 exp Animals/ not Humans/ (17546303)
110 108 not 109 [ANIMAL‐ONLY REMOVED] (13985)
111 (comment or editorial or news or newspaper article).pt. (1896536)
112 (letter not (letter and randomised controlled trial)).pt. (2072075)
113 110 not (111 or 112) [OPINION PIECES REMOVED] (13938)
114 113 use medall [MEDLINE RECORDS] (6474)
115 empowerment/ (27108)
116 attitude/ (107645)
117 attitude to health/ (190744)
118 attitude to life/ (657)
119 maternal attitude/ (2423)
120 attitude to pregnancy/ (580)
121 social attitude/ (2312)
122 ((attitud* or opinion*) adj3 (diet* or eating or feeding or food? or health* or malnutrit* or malnourish* or meal? or nutrient? or nutrition or nutritive or snack?)).tw,kw. (45396)
123 behavior/ (209991)
124 ((intention? or intent? or intend*) adj3 (diet* or eating or feeding or food? or health* or malnutrit* or malnourish* or meal? or nutrient? or nutrition or nutritive or snack?)).tw,kw. (9069)
125 motivation/ (216063)
126 (motivat* adj3 (diet* or eating or feeding or food? or health* or malnutrit* or malnourish* or meal? or nutrient? or nutrition or nutritive or snack?)).tw,kw. (14513)
127 (encourag* adj3 (diet* or eating or feeding or food? or health* or malnutrit* or malnourish* or meal? or nutrient? or nutrition or nutritive or snack?)).tw,kw. (14391)
128 personal autonomy/ (29331)
129 exp self concept/ (373341)
130 (capable or capabilit* or emancipat* or empower* or agency or self‐efficac* or power* or autonomy or (self adj2 determin*) or (self adj2 concept*) or (self adj2 confiden*) or (self adj2 percept*) or (self adj2 esteem)).tw,kw. (2512016)
131 (abilit* adj3 (act or acted or acting or acts or action?)).tw,kw. (7289)
132 women's rights/ (15820)
133 ((wom#n* adj2 right?) or (wom#n* adj2 status*) or (girl* adj2 right?) or (girl* adj2 status*) or (female? adj2 right?) or (female? adj2 status*) or (wife adj2 right?) or (wife adj2 status*) or (mother* adj2 right?) or (mother* adj2 status*) or (wives adj2 right?) or (wives adj2 status*)).tw,kw. (36781)
134 ((wom#n* or girl* or female? or mother* or wife or wives) adj2 (engag* or involv* or participat*)).tw,kw. (74297)
135 (active$2 adj2 (engag* or involv* or participat*)).tw,kw. (61734)
136 participatory*.tw,kw. (36423)
137 ((wom#n* or girl* or female? or mother* or wife or wives) adj3 (advoca* or input* or ombuds* or represent*)).tw,kw. (21169)
138 ((citizen* or communit* or gender* or public or village?) adj3 (advoca* or input* or ombuds* or represent*)).tw,kw. (31657)
139 gender identity/ (41227)
140 ((wom#n* or girl* or female? or mother* or wife or wives) adj3 role?).tw,kw. (28723)
141 social norm/ (10891)
142 ((social* or societ* or cultural* or group?) adj3 (custom* or norm?)).tw,kw. (36159)
143 or/115–142 (3718206)
144 nutrition/ (124564)
145 nutritional health/ (6190)
146 exp nutritional requirement/ (39461)
147 exp nutritional status/ (106035)
148 nutritional value/ (31912)
149 adolescent nutrition/ (202)
150 maternal nutrition/ (11173)
151 malnutrition/ (75398)
152 exp nutritional deficiency/ (283978)
153 exp protein deficiency/ (44783)
154 (avitaminos#s or hypovitaminos#s or hypo‐vitaminos#s).tw,kw. (8961)
155 ((ascorbic acid? or ferrous ascorbate or hybrin or magnesium ascorbate or magnesium ascorbicum or magnesium di‐L‐ascorbate or magnorbin or sodium ascorbate) adj3 (deficien* or deficit? or inadequa* or insufficien* or intak* or lack* or supplement*)).tw,kw. (4087)
156 scurvy.tw,kw. (4353)
157 ((all‐trans‐retinol or aquasol a or retinol) adj3 (deficien* or deficit? or inadequa* or insufficien* or intak* or lack* or supplement*)).tw,kw. (2055)
158 ((bursine or choline or fagine or vagine) adj3 (deficien* or deficit? or inadequa* or insufficien* or intak* or lack* or supplement*)).tw,kw. (7851)
159 ((folacin or folate or folic acid? or folvite or IFA or pteroylglutamic acid?) adj3 (deficien* or deficit? or inadequa* or insufficien* or intak* or lack*or supplement*)).tw,kw. (17918)
160 (hyperhomocystein?emi* or hyper‐homocystein?emi*).tw,kw. (15531)
161 ((niacin? or enduracin or induracin or lithium nicotinate or nicamin or nico‐400 or nicobid or nicocap or nicolar or nicotinate or nicotinic acid? or wampocap) adj3 (deficien* or deficit? or inadequa* or insufficien* or intak* or lack* or supplement*)).tw,kw. (1816)
162 ((tryptophan or ardeydorm or ardeytropin or L‐tryptophan or levotryptophan or lyphan or naturruhe or optimax or pms‐tryptophan or trofan or tryptacin or tryptan or ratio‐tryptophan) adj3 (deficien* or deficit? or inadequa* or insufficien* or intak* or lack* or supplement*)).tw,kw. (2805)
163 pellagra.tw,kw. (2772)
164 (riboflavin? adj3 (deficien* or deficit? or inadequa* or insufficien* or intak* or lack* or supplement*)).tw,kw. (3220)
165 ((thiamine? or aneurin or thiamin) adj3 (deficien* or deficit? or inadequa* or insufficien* or intak* or lack* or supplement*)).tw,kw. (7929)
166 (pyridoxine? adj3 (deficien* or deficit? or inadequa* or insufficien* or intak* or lack* or supplement*)).tw,kw. (2690)
167 exp overnutrition/ (694990)
168 growth disorder/ (33286)
169 stunting/ (19313)
170 ((growth adj2 stunt$2) or stunting).tw,kw. (14278)
171 (nutrient? or nutrition* or nutritive or nourish* or malnutrit* or malnourish* or undernutrition or undernourish* or overnutrition or overnourish* or obese or obesity or wasting or underweight or under weight or overweight or over weight).tw,kw. (1793960)
172 anemia/ (242868)
173 iron deficiency anemia/ (37358)
174 (an?emic or an?emia* or chloros#s).tw,kw. (395051)
175 eating habit/ (99906)
176 feeding behavior/ (172106)
177 ((eating or feeding or food or meal? or snack*) adj3 (behavio?r* or habit? or habitual* or pattern* or practice?)).tw,kw. (144241)
178 diet/ (402699)
179 (diet or dieted or diets or dietary or dieting).tw,kw. (1187821)
180 caloric intake/ (96310)
181 ((calori* or energy or protein?) adj3 intak*).tw,kw. (117328)
182 dietary intake/ (73687)
183 healthy diet/ (4701)
184 ((eating or feeding or food? or meal? or snack*) adj3 (healthy or unhealthy)).tw,kw. (39335)
185 exp nutrient/ (677172)
186 nutrient availability/ (3222)
187 nutrient content/ (5017)
188 nutrient limitation/ (1029)
189 nutrient supply/ (2308)
190 nutrient uptake/ (2874)
191 (micronutri* or micro‐nutri*).tw,kw. (36132)
192 exp trace element/ (366886)
193 (trace element? or trace mineral? or micromineral? or micro‐mineral?).tw,kw. (46036)
194 chromium/ (59565)
195 copper/ (178475)
196 iodine/ (75382)
197 manganese/ (74289)
198 molybdenum/ (21257)
199 selenium/ (58025)
200 zinc/ (170532)
201 (calcium or chromium or copper or fluoride or iodine or iron or manganese or molybdenum or selenium or zinc).tw,kw. (1949030)
202 vitamin/ (89429)
203 vitamin*.tw,kw. (522115)
204 dietary supplement/ (61637)
205 ((eating or feeding or food? or meal? or snack*) adj3 supplement*).tw,kw. (26015)
206 nutraceutical/ (64077)
207 n?utr#ceutical?.tw,kw. (14562)
208 fortified food/ (11096)
209 ((fortif* or enrich* or functional) adj2 (eating or feeding or food? or meal? or snack*)).tw,kw. (25657)
210 ((home? or school* or work*) adj2 (ate or eaten or eating or eats or breakfast* or lunch* or dinner* or supper* or feed* or food? or meal?)).tw,kw. (20058)
211 food availability/ (3871)
212 ((eating or feeding or food? or meal? or snack*) adj3 (secure* or securit* or insecure* or insecurit* or supply* or supplie*)).tw,kw. (31599)
213 or/144–212 (6081563)
214 143 and 213 (313876)
215 developing country/ (165162)
216 (Africa or Asia or Caribbean or West Indies or South America or Latin America or Central America).hw,tw,kw. (640442)
217 (Afghanistan or Albania or Algeria or Angola or Argentina or Armenia or Armenian or Azerbaijan or Bangladesh or Benin or Byelarus or Byelorussian or Belarus or Belorussian or Belorussia or Belize or Bhutan or Bolivia or Bosnia or Herzegovina or Hercegovina or Botswana or Brazil or Bulgaria or Burkina Faso or Burkina Fasso or Upper Volta or Burundi or Urundi or Cambodia or Khmer Republic or Kampuchea or Cameroon or Cameroons or Cameron or Camerons or Cape Verde or Central African Republic or Chad or China or Colombia or Comoros or Comoro Islands or Comores or Mayotte or Congo or Zaire or Costa Rica or Cote d'Ivoire or Ivory Coast or Cuba or Djibouti or French Somaliland or Dominica or Dominican Republic or East Timor or East Timur or Timor Leste or Ecuador or Egypt or United Arab Republic or El Salvador or Eritrea or Ethiopia or Fiji or Gabon or Gabonese Republic or Gambia or Gaza or Georgia Republic or Georgian Republic or Ghana or Grenada or Guatemala or Guinea or Guiana or Guyana or Haiti or Honduras or India or Maldives or Indonesia or Iran or Iraq or Jamaica or Jordan or Kazakhstan or Kazakh or Kenya or Kiribati or Korea or Kosovo or Kyrgyzstan or Kirghizia or Kyrgyz Republic or Kirghiz or Kirgizstan or Lao PDR or Laos or Lebanon or Lesotho or Basutoland or Liberia or Libya or Macedonia or Madagascar or Malagasy Republic or Malaysia or Malaya or Malay or Sabah or Sarawak or Malawi or Mali or Marshall Islands or Mauritania or Mauritius or Agalega Islands or Mexico or Micronesia or Middle East or Moldova or Moldovia or Moldovian or Mongolia or Montenegro or Morocco or Ifni or Mozambique or Myanmar or Myanma or Burma or Namibia or Nepal or Netherlands Antilles or Nicaragua or Niger or Nigeria or Muscat or Pakistan or Palau or Palestine or Panama or Paraguay or Peru or Philippines or Philipines or Phillipines or Phillippines or Papua New Guinea or Romania or Rumania or Roumania or Rwanda or Ruanda or Saint Lucia or St Lucia or Saint Vincent or St Vincent or Grenadines or Samoa or Samoan Islands or Navigator Island or Navigator Islands or Sao Tome or Senegal or Serbia or Montenegro or Seychelles or Sierra Leone or Sri Lanka or Solomon Islands or Somalia or Sudan or Suriname or Surinam or Swaziland or South Africa or Syria or Tajikistan or Tadzhikistan or Tadjikistan or Tadzhik or Tanzania or Thailand or Togo or Togolese Republic or Tonga or Tunisia or Turkey or Turkmenistan or Turkmen or Uganda or Ukraine or Uzbekistan or Uzbek or Vanuatu or New Hebrides or Venezuela or Vietnam or Viet Nam or West Bank or Yemen or Zambia or Zimbabwe).hw,tw,kw. (3320872)
218 exp africa/ or exp africa, northern/ or algeria/ or egypt/ or libya/ or morocco/ or tunisia/ or exp “africa south of the sahara”/ or africa, central/ or cameroon/ or central african republic/ or chad/ or congo/ or “democratic republic of the congo”/ or equatorial guinea/ or gabon/ or africa, eastern/ or burundi/ or djibouti/ or eritrea/ or ethiopia/ or kenya/ or rwanda/ or somalia/ or south sudan/ or sudan/ or tanzania/ or uganda/ or africa, southern/ or angola/ or botswana/ or lesotho/ or malawi/ or mozambique/ or namibia/ or south africa/ or swaziland/ or zambia/ or zimbabwe/ or africa, western/ or benin/ or burkina faso/ or cape verde/ or cote d'ivoire/ or gambia/ or ghana/ or guinea/ or guinea‐bissau/ or liberia/ or mali/ or mauritania/ or niger/ or nigeria/ or senegal/ or sierra leone/ or togo/ or americas/ or exp caribbean region/ or exp west indies/ or exp central america/ or belize/ or costa rica/ or el salvador/ or guatemala/ or honduras/ or nicaragua/ or panama/ or panama canal zone/ or latin america/ or mexico/ or exp south america/ or argentina/ or bolivia/ or brazil/ or chile/ or colombia/ or ecuador/ or french guiana/ or guyana/ or paraguay/ or peru/ or suriname/ or uruguay/ or venezuela/ or asia/ or asia, central/ or kazakhstan/ or kyrgyzstan/ or tajikistan/ or turkmenistan/ or uzbekistan/ or exp asia, southeastern/ or borneo/ or brunei/ or cambodia/ or timor‐leste/ or indonesia/ or laos/ or malaysia/ or mekong valley/ or myanmar/ or philippines/ or singapore/ or thailand/ or vietnam/ or asia, western/ or bangladesh/ or bhutan/ or india/ or sikkim/ or middle east/ or afghanistan/ or bahrain/ or iran/ or iraq/ or israel/ or jordan/ or kuwait/ or lebanon/ or oman/ or qatar/ or saudi arabia/ or syria/ or turkey/ or united arab emirates/ or yemen/ or nepal/ or pakistan/ or sri lanka/ or far east/ or china/ or beijing/ or macau/ or tibet/ or korea/ or mongolia/ or taiwan/ or indian ocean islands/ or comoros/ or madagascar/ or mauritius/ or reunion/ or seychelles/ or pacific islands/ or exp melanesia/ or exp micronesia/ or polynesia/ or pitcairn island/ or exp samoa/ or tonga/ or prince edward island/ or west indies/ or “antigua and barbuda”/ or bahamas/ or barbados/ or cuba/ or dominica/ or dominican republic/ or grenada/ or guadeloupe/ or haiti/ or jamaica/ or martinique/ or netherlands antilles/ or puerto rico/ or “saint kitts and nevis”/ or saint lucia/ or “saint vincent and the grenadines”/ or “trinidad and tobago”/ or united states virgin islands/ or oceania/ (2424512)
219 ((developing or less* developed or under developed or underdeveloped or middle income or low* income or underserved or under served or deprived or poor*) adj (countr* or nation? or population? or region? or world or state*)).tw,kw. (237596)
220 ((developing or less* developed or under developed or underdeveloped or middle income or low* income or poor*) adj econom*).tw,kw. (3036)
221 (low* adj (gdp or gnp or gross domestic or gross national)).tw,kw. (645)
222 (low adj3 middle adj3 countr*).tw,kw. (31748)
223 (lmic or lmics or third world or lami countr*).tw,kw. (16181)
224 transitional countr*.tw,kw. (462)
225 or/215–224 (4044608)
226 214 and 225 (36165)
227 exp named groups by pregnancy/ (101747)
228 pregnant woman/ (82942)
229 female/ and (adult/ or young adult/ or juvenile/ or adolescent/ or adolescent parent/) (9605834)
230 (woman* or women* or matern* or mother* or wife or wives or pregnan*).tw,kw. (4340623)
231 ((female? or girl*) adj5 (adolescen* or adult* or child‐bearing or childbearing or highschool* or school* or teen* or marry* or marriage* or marrie? or menstru* or post‐pubescen* or postpubescen* or post‐puberty or postpuberty or reproductive or spous* or youth* or young adult?)).tw,kw. (292884)
232 ((female? or girl or girls) adj2 (ten year? or ten yr? or 10 year? or 10 yr? or eleven year? or eleven yr? or 11 year? or 11 yr? or twelve year? or twelve yr? or 12 year? or 12 yr?)).tw,kw. (28995)
233 ((female? or girl or girls) adj2 (age? or old) adj3 (“10” or ten or “11” or eleven or “12” or twelve)).tw,kw. (66013)
234 young girl?.tw,kw. (10038)
235 or/227–234 (12506222)
236 226 and 235 (20195)
237 exp animal/ or exp animal experimentation/ or exp animal model/ or exp animal experiment/ or nonhuman/ or exp vertebrate/ (50805119)
238 exp human/ or exp human experimentation/ or exp human experiment/ (38981083)
239 237 not 238 (11825722)
240 236 not 239 [ANIMAL‐ONLY REMOVED] (20036)
241 editorial.pt. (1081172)
242 letter.pt. not (letter.pt. and randomised controlled trial/) (2071940)
243 240 not (241 or 242) [OPINION PIECES REMOVED] (19985)
244 243 use emczd [EMBASE RECORDS] (9903)
245 Social Identity/ (105394)
246 Group Identity/ (1322)
247 Attitudes/ (72109)
248 Community Attitudes/ (2559)
249 Death Attitudes/ (19039)
250 Eating Attitudes/ (1539)
251 Female Attitudes/ (1625)
252 Health Attitudes/ (94051)
253 exp Sex Role Attitudes/ (12044)
254 ((attitud* or opinion*) adj3 (diet* or eating or feeding or food? or health* or malnutrit* or malnourish* or meal? or nutrient? or nutrition or nutritive or snack?)).tw. (41335)
255 exp Intention/ (3953989)
256 ((intention? or intent? or intend*) adj3 (diet* or eating or feeding or food? or health* or malnutrit* or malnourish* or meal? or nutrient? or nutrition or nutritive or snack?)).tw. (8802)
257 Motivation/ (216063)
258 Expectations/ (81852)
259 (motivat* adj3 (diet* or eating or feeding or food? or health* or malnutrit* or malnourish* or meal? or nutrient? or nutrition or nutritive or snack?)).tw. (13785)
260 (encourag* adj3 (diet* or eating or feeding or food? or health* or malnutrit* or malnourish* or meal? or nutrient? or nutrition or nutritive or snack?)).tw. (14391)
261 Self‐Efficacy/ (92885)
262 "Independence (Personality)"/ (4988)
263 Self‐Determination/ (20619)
264 Self‐Concept/ (184924)
265 Self‐Confidence/ (57437)
266 Self‐Esteem/ (101452)
267 (capable or capabilit* or emancipat* or empower* or agency or self‐efficac* or power* or autonomy or (self adj2 determin*) or (self adj2 concept*) or (self adj2 confiden*) or (self adj2 percept*) or (self adj2 esteem)).tw. (2502151)
268 (abilit* adj3 (act or acted or acting or acts or action?)).tw. (7288)
269 Self‐Perception/ (146381)
270 ((wom#n* adj2 right?) or (wom#n* adj2 status*) or (girl* adj2 right?) or (girl* adj2 status*) or (female? adj2 right?) or (female? adj2 status*) or (wife adj2 right?) or (wife adj2 status*) or (mother* adj2 right?) or (mother* adj2 status*) or (wives adj2 right?) or (wives adj2 status*)).tw. (34478)
271 Involvement/ (6748)
272 Participation/ (7668)
273 ((wom#n* or girl* or female? or mother* or wife or wives) adj2 (engag* or involv* or participat*)).tw. (74248)
274 (active$2 adj2 (engag* or involv* or participat*)).tw. (61718)
275 participatory*.tw. (35257)
276 ((wom#n* or girl* or female? or mother* or wife or wives) adj3 (advoca* or input* or ombuds* or represent*)).tw. (21143)
277 ((citizen* or communit* or gender* or public or village?) adj3 (advoca* or input* or ombuds* or represent*)).tw. (31568)
278 Gender Equality/ (667)
279 Role Expectations/ (1621)
280 Sex Roles/ (34691)
281 ((wom#n* or girl* or female? or mother* or wife or wives) adj3 role?).tw. (28694)
282 Social Norms/ (10338)
283 ((social* or societ* or cultural* or group?) adj3 (custom* or norm?)).tw. (35820)
284 or/245–283 (6964179)
285 Nutrition/ (124564)
286 exp Nutritional Deficiencies/ (124075)
287 (avitaminos#s or hypovitaminos#s or hypo‐vitaminos#s).tw. (8621)
288 ((ascorbic acid? or ferrous ascorbate or hybrin or magnesium ascorbate or magnesium ascorbicum or magnesium di‐L‐ascorbate or magnorbin or sodium ascorbate) adj3 (deficien* or deficit? or inadequa* or insufficien* or intak* or lack* or supplement*)).tw. (3989)
289 scurvy.tw. (3901)
290 ((all‐trans‐retinol or aquasol a or retinol) adj3 (deficien* or deficit? or inadequa* or insufficien* or intak* or lack* or supplement*)).tw. (1959)
291 ((bursine or choline or fagine or vagine) adj3 (deficien* or deficit? or inadequa* or insufficien* or intak* or lack* or supplement*)).tw. (7797)
292 ((folacin or folate or folic acid? or folvite or IFA or pteroylglutamic acid?) adj3 (deficien* or deficit? or inadequa* or insufficien* or intak* or lack*or supplement*)).tw. (17757)
293 (hyperhomocystein?emi* or hyper‐homocystein?emi*).tw. (15197)
294 ((niacin? or enduracin or induracin or lithium nicotinate or nicamin or nico‐400 or nicobid or nicocap or nicolar or nicotinate or nicotinic acid? or wampocap) adj3 (deficien* or deficit? or inadequa* or insufficien* or intak* or lack* or supplement*)).tw. (1792)
295 ((tryptophan or ardeydorm or ardeytropin or l‐tryptophan or levotryptophan or lyphan or naturruhe or optimax or pms‐tryptophan or trofan or tryptacin or tryptan or ratio‐tryptophan) adj3 (deficien* or deficit? or inadequa* or insufficien* or intak* or lack* or supplement*)).tw. (2784)
296 pellagra.tw. (2584)
297 (riboflavin? adj3 (deficien* or deficit? or inadequa* or insufficien* or intak* or lack* or supplement*)).tw. (3196)
298 ((thiamine? or aneurin or thiamin) adj3 (deficien* or deficit? or inadequa* or insufficien* or intak* or lack* or supplement*)).tw. (7817)
299 (pyridoxine? adj3 (deficien* or deficit? or inadequa* or insufficien* or intak* or lack* or supplement*)).tw. (2639)
300 Obesity/ (598386)
301 ((growth adj2 stunt$2) or stunting).tw. (13942)
302 (nutrient? or nutrition* or nutritive or nourish* or malnutrit* or malnourish* or undernutrition or undernourish* or overnutrition or overnourish* or obese or obesity or wasting or underweight or under weight or overweight or over weight).tw. (1748835)
303 Anemia/ (242868)
304 (an?emic or an?emia* or chloros#s).tw. (380030)
305 Eating Behavior/ (174683)
306 ((eating or feeding or food or meal? or snack*) adj3 (behavio?r* or habit? or habitual* or pattern* or practice?)).tw. (141008)
307 Diets/ (161532)
308 (diet or dieted or diets or dietary or dieting).tw. (1165001)
309 Food Intake/ (184009)
310 ((calori* or energy or protein?) adj3 intak*).tw. (115118)
311 ((eating or feeding or food? or meal? or snack*) adj3 (healthy or unhealthy)).tw. (39122)
312 (micronutri* or micro‐nutri*).tw. (35029)
313 Metallic Elements/ (436)
314 (trace element? or trace mineral? or micromineral? or micro‐mineral?).tw. (43646)
315 exp Calcium/ (570689)
316 Copper/ (178475)
317 Iron/ (259984)
318 Zinc/ (170532)
319 (calcium or chromium or copper or fluoride or iodine or iron or manganese or molybdenum or selenium or zinc).tw. (1900718)
320 Vitamins/ (89507)
321 vitamin*.tw. (499855)
322 Dietary Supplements/ (67852)
323 ((eating or feeding or food? or meal? or snack*) adj3 supplement*).tw. (25555)
324 n?utr#ceutical?.tw. (13721)
325 ((fortif* or enrich* or functional) adj2 (eating or feeding or food? or meal? or snack*)).tw. (24593)
326 ((home? or school* or work*) adj2 (ate or eaten or eating or eats or breakfast* or lunch* or dinner* or supper* or feed* or food? or meal?)).tw. (19806)
327 Food Deprivation/ (18608)
328 ((eating or feeding or food? or meal? or snack*) adj3 (secure* or securit* or insecure* or insecurit* or supply* or supplie*)).tw. (30873)
329 or/285–328 (5847359)
330 284 and 329 (656017)
331 Developing Countries/ (159704)
332 (Africa? or Asia? or Caribbean or West Indies or South America? or Latin America? or Central America?).tw. (924816)
333 (Afghanistan or Albania or Algeria or Angola or Argentina or Armenia or Armenian or Azerbaijan or Bangladesh or Benin or Byelarus or Byelorussian or Belarus or Belorussian or Belorussia or Belize or Bhutan or Bolivia or Bosnia or Herzegovina or Hercegovina or Botswana or Brazil or Bulgaria or Burkina Faso or Burkina Fasso or Upper Volta or Burundi or Urundi or Cambodia or Khmer Republic or Kampuchea or Cameroon or Cameroons or Cameron or Camerons or Cape Verde or Central African Republic or Chad or China or Colombia or Comoros or Comoro Islands or Comores or Mayotte or Congo or Zaire or Costa Rica or Cote d'Ivoire or Ivory Coast or Cuba or Djibouti or French Somaliland or Dominica or Dominican Republic or East Timor or East Timur or Timor Leste or Ecuador or Egypt or United Arab Republic or El Salvador or Eritrea or Ethiopia or Fiji or Gabon or Gabonese Republic or Gambia or Gaza or Georgia Republic or Georgian Republic or Ghana or Grenada or Guatemala or Guinea or Guiana or Guyana or Haiti or Honduras or India or Maldives or Indonesia or Iran or Iraq or Jamaica or Jordan or Kazakhstan or Kazakh or Kenya or Kiribati or Korea or Kosovo or Kyrgyzstan or Kirghizia or Kyrgyz Republic or Kirghiz or Kirgizstan or Lao PDR or Laos or Lebanon or Lesotho or Basutoland or Liberia or Libya or Macedonia or Madagascar or Malagasy Republic or Malaysia or Malaya or Malay or Sabah or Sarawak or Malawi or Mali or Marshall Islands or Mauritania or Mauritius or Agalega Islands or Mexico or Micronesia or Middle East or Moldova or Moldovia or Moldovian or Mongolia or Montenegro or Morocco or Ifni or Mozambique or Myanmar or Myanma or Burma or Namibia or Nepal or Netherlands Antilles or Nicaragua or Niger or Nigeria or Muscat or Pakistan or Palau or Palestine or Panama or Paraguay or Peru or Philippines or Philipines or Phillipines or Phillippines or Papua New Guinea or Romania or Rumania or Roumania or Rwanda or Ruanda or Saint Lucia or St Lucia or Saint Vincent or St Vincent or Grenadines or Samoa or Samoan Islands or Navigator Island or Navigator Islands or Sao Tome or Senegal or Serbia or Montenegro or Seychelles or Sierra Leone or Sri Lanka or Solomon Islands or Somalia or Sudan or Suriname or Surinam or Swaziland or South Africa or Syria or Tajikistan or Tadzhikistan or Tadjikistan or Tadzhik or Tanzania or Thailand or Togo or Togolese Republic or Tonga or Tunisia or Turkey or Turkmenistan or Turkmen or Uganda or Ukraine or Uzbekistan or Uzbek or Vanuatu or New Hebrides or Venezuela or Vietnam or Viet Nam or West Bank or Yemen or Zambia or Zimbabwe).tw. (2449161)
334 ((developing or less* developed or under developed or underdeveloped or middle income or low* income or underserved or under served or deprived or poor*) adj (countr* or nation? or population? or region? or world or state*)).tw. (233745)
335 ((developing or less* developed or under developed or underdeveloped or middle income or low* income or poor*) adj econom*).tw. (3024)
336 (low* adj (gdp or gnp or gross domestic or gross national)).tw. (641)
337 (low adj3 middle adj3 countr*).tw. (31256)
338 (lmic or lmics or third world or lami countr*).tw. (15802)
339 transitional countr*.tw. (456)
340 or/331–339 (3253265)
341 330 and 340 (74884)
342 Adolescent Mothers/ (2282)
343 Expectant Mothers/ (637)
344 (woman* or women* or matern* or mother* or wife or wives or pregnan*).tw. (4311022)
345 ((female? or girl*) adj5 (adolescen* or adult* or child‐bearing or childbearing or highschool* or school* or teen* or marry* or marriage* or marrie? or menstru* or post‐pubescen* or postpubescen* or post‐puberty or postpuberty or reproductive or spous* or youth* or young adult?)).tw. (285627)
346 ((female? or girl or girls) adj2 (ten year? or ten yr? or 10 year? or 10 yr? or eleven year? or eleven yr? or 11 year? or 11 yr? or twelve year? or twelve yr? or 12 year? or 12 yr?)).tw. (28995)
347 ((female? or girl or girls) adj2 (age? or old) adj3 ("10" or ten or "11" or eleven or "12" or twelve)).tw. (66012)
348 young girl?.tw. (10026)
349 or/342–348 (4596550)
350 Human Females/ (88344)
351 Wives/ (13136)
352 350 or 351 (101298)
353 limit 352 to (200 adolescence <age 13 to 17 yrs> or 320 young adulthood <age 18 to 29 yrs> or 340 thirties <age 30 to 39 yrs> or 360 middle age <age 40 to 64 yrs>) [Limit not valid in Embase,Ovid MEDLINE(R),Ovid MEDLINE(R) Daily Update,Ovid MEDLINE(R) In‐Process,Ovid MEDLINE(R) Publisher,CCTR,CDSR,DARE,CLHTA,CLEED; records were retained] (43270)
354 349 or 353 (4605043)
355 341 and 354 (27908)
356 355 use medall,emczd,cctr,coch,dare,clhta,cleed (26667)
357 355 not 356 [PSYCINFO RECORDS] (1241)
358 “Power (Psychology)”/ (158608)
359 Attitude/ (107645)
360 Attitude to Health/ (190744)
361 ((attitud* or opinion*) adj3 (diet* or eating or feeding or food? or health* or malnutrit* or malnourish* or meal? or nutrient? or nutrition or nutritive or snack?)).ti,ab,kw. (42279)
362 Intention/ (171233)
363 ((intention? or intent? or intend*) adj3 (diet* or eating or feeding or food? or health* or malnutrit* or malnourish* or meal? or nutrient? or nutrition or nutritive or snack?)).ti,ab,kw. (8886)
364 Motivation/ (216063)
365 (motivat* adj3 (diet* or eating or feeding or food? or health* or malnutrit* or malnourish* or meal? or nutrient? or nutrition or nutritive or snack?)).ti,ab,kw. (14263)
366 (encourag* adj3 (diet* or eating or feeding or food? or health* or malnutrit* or malnourish* or meal? or nutrient? or nutrition or nutritive or snack?)).ti,ab,kw. (14226)
367 Self Efficacy/ (92885)
368 Personal Autonomy/ (29331)
369 Self Concept/ (184924)
370 (capable or capabilit* or emancipat* or empower* or agency or self‐efficac* or power* or autonomy or (self adj2 determin*) or (self adj2 concept*) or (self adj2 confiden*) or (self adj2 percept*) or (self adj2 esteem)).ti,ab,kw. (2484054)
371 (abilit* adj3 (act or acted or acting or acts or action?)).ti,ab,kw. (7236)
372 Women's Rights/ (15820)
373 ((wom#n* adj2 right?) or (wom#n* adj2 status*) or (girl* adj2 right?) or (girl* adj2 status*) or (female? adj2 right?) or (female? adj2 status*) or (wife adj2 right?) or (wife adj2 status*) or (mother* adj2 right?) or (mother* adj2 status*) or (wives adj2 right?) or (wives adj2 status*)).ti,ab,kw. (36005)
374 ((wom#n* or girl* or female? or mother* or wife or wives) adj2 (engag* or involv* or participat*)).ti,ab,kw. (73351)
375 (active$2 adj2 (engag* or involv* or participat*)).ti,ab,kw. (61322)
376 participatory*.ti,ab,kw. (35693)
377 ((wom#n* or girl* or female? or mother* or wife or wives) adj3 (advoca* or input* or ombuds* or represent*)).ti,ab,kw. (20803)
378 ((citizen* or communit* or gender* or public or village?) adj3 (advoca* or input* or ombuds* or represent*)).ti,ab,kw. (31346)
379 Gender Identity/ (41227)
380 ((wom#n* or girl* or female? or mother* or wife or wives) adj3 role?).ti,ab,kw. (27434)
381 Social Norms/ (10338)
382 ((social* or societ* or cultural* or group?) adj3 (custom* or norm?)).ti,ab,kw. (34970)
383 or/358–382 (3552165)
384 Nutritional Requirements/ (37442)
385 Nutritional Status/ (105385)
386 Nutritive Value/ (30809)
387 Adolescent Nutritional Physiological Phenomena/ (1838)
388 Maternal Nutritional Physiological Phenomena/ (13721)
389 Malnutrition/ (75398)
390 exp Deficiency Diseases/ (260675)
391 (avitaminos#s or hypovitaminos#s or hypo‐vitaminos#s).ti,ab,kw. (8948)
392 ((ascorbic acid? or ferrous ascorbate or hybrin or magnesium ascorbate or magnesium ascorbicum or magnesium di‐L‐ascorbate or magnorbin or sodium ascorbate) adj3 (deficien* or deficit? or inadequa* or insufficien* or intak* or lack* or supplement*)).ti,ab,kw. (4074)
393 scurvy.ti,ab,kw. (4341)
394 ((all‐trans‐retinol or aquasol a or retinol) adj3 (deficien* or deficit? or inadequa* or insufficien* or intak* or lack* or supplement*)).ti,ab,kw. (2037)
395 ((bursine or choline or fagine or vagine) adj3 (deficien* or deficit? or inadequa* or insufficien* or intak* or lack* or supplement*)).ti,ab,kw. (7843)
396 ((folacin or folate or folic acid? or folvite or IFA or pteroylglutamic acid?) adj3 (deficien* or deficit? or inadequa* or insufficien* or intak* or lack*or supplement*)).ti,ab,kw. (17838)
397 (hyperhomocystein?emi* or hyper‐homocystein?emi*).ti,ab,kw. (15508)
398 ((niacin? or enduracin or induracin or lithium nicotinate or nicamin or nico‐400 or nicobid or nicocap or nicolar or nicotinate or nicotinic acid? or wampocap) adj3 (deficien* or deficit? or inadequa* or insufficien* or intak* or lack* or supplement*)).ti,ab,kw. (1808)
399 ((tryptophan or ardeydorm or ardeytropin or l‐tryptophan or levotryptophan or lyphan or naturruhe or optimax or pms‐tryptophan or trofan or tryptacin or tryptan or ratio‐tryptophan) adj3 (deficien* or deficit? or inadequa* or insufficien* or intak* or lack* or supplement*)).ti,ab,kw. (2791)
400 pellagra.ti,ab,kw. (2759)
401 (riboflavin? adj3 (deficien* or deficit? or inadequa* or insufficien* or intak* or lack* or supplement*)).ti,ab,kw. (3214)
402 ((thiamine? or aneurin or thiamin) adj3 (deficien* or deficit? or inadequa* or insufficien* or intak* or lack* or supplement*)).ti,ab,kw. (7904)
403 (pyridoxine? adj3 (deficien* or deficit? or inadequa* or insufficien* or intak* or lack* or supplement*)).ti,ab,kw. (2684)
404 Overnutrition/ (5853)
405 exp Obesity/ (711110)
406 Growth Disorders/ (26038)
407 ((growth adj2 stunt$2) or stunting).ti,ab,kw. (14204)
408 (nutrient? or nutrition* or nutritive or nourish* or malnutrit* or malnourish* or undernutrition or undernourish* or overnutrition or overnourish* or obese or obesity or wasting or underweight or under weight or overweight or over weight).ti,ab,kw. (1787088)
409 Anemia/ (242868)
410 exp Anemia, Hypochromic/ (46742)
411 (an?emic or an?emia* or chloros#s).ti,ab,kw. (393916)
412 Feeding Behavior/ (172106)
413 ((eating or feeding or food or meal? or snack*) adj3 (behavio?r* or habit? or habitual* or pattern* or practice?)).ti,ab,kw. (141188)
414 Diet/ (402699)
415 (diet or dieted or diets or dietary or dieting).ti,ab,kw. (1184491)
416 Energy Intake/ (93531)
417 ((calori* or energy or protein?) adj3 intak*).ti,ab,kw. (116855)
418 Healthy Diet/ (4701)
419 ((eating or feeding or food? or meal? or snack*) adj3 (healthy or unhealthy)).ti,ab,kw. (39049)
420 exp Micronutrients/ (688381)
421 (micronutri* or micro‐nutri*).ti,ab,kw. (35918)
422 (trace element? or trace mineral? or micromineral? or micro‐mineral?).ti,ab,kw. (45946)
423 Calcium, Dietary/ (31557)
424 Chromium/ (59565)
425 Copper/ (178475)
426 Iodine/ (75382)
427 Iron/ (259984)
428 Manganese/ (74289)
429 Molybdenum/ (21257)
430 Selenium/ (58025)
431 Zinc/ (170532)
432 (calcium or chromium or copper or fluoride or iodine or iron or manganese or molybdenum or selenium or zinc).ti,ab,kw. (1944286)
433 vitamin*.ti,ab,kw. (520663)
434 exp Dietary Supplements/ (89126)
435 ((eating or feeding or food? or meal? or snack*) adj3 supplement*).ti,ab,kw. (25760)
436 n?utr#ceutical?.ti,ab,kw. (14503)
437 Food, Fortified/ (11093)
438 ((fortif* or enrich* or functional) adj2 (eating or feeding or food? or meal? or snack*)).ti,ab,kw. (25516)
439 ((home? or school* or work*) adj2 (ate or eaten or eating or eats or breakfast* or lunch* or dinner* or supper* or feed* or food? or meal?)).ti,ab,kw. (19800)
440 Food Supply/ (28920)
441 ((eating or feeding or food? or meal? or snack*) adj3 (secure* or securit* or insecure* or insecurit* or supply* or supplie*)).ti,ab,kw. (31461)
442 or/384–441 (6067800)
443 383 and 442 (295635)
444 Developing Countries/ (159704)
445 Africa/ or Asia/ or Caribbean/ or West Indies/ or South America/ or Latin America/ or Central America/ (228463)
446 (Afghanistan or Albania or Algeria or Angola or Argentina or Armenia or Armenian or Azerbaijan or Bangladesh or Benin or Byelarus or Byelorussian or Belarus or Belorussian or Belorussia or Belize or Bhutan or Bolivia or Bosnia or Herzegovina or Hercegovina or Botswana or Brazil or Bulgaria or Burkina Faso or Burkina Fasso or Upper Volta or Burundi or Urundi or Cambodia or Khmer Republic or Kampuchea or Cameroon or Cameroons or Cameron or Camerons or Cape Verde or Central African Republic or Chad or China or Colombia or Comoros or Comoro Islands or Comores or Mayotte or Congo or Zaire or Costa Rica or Cote d'Ivoire or Ivory Coast or Cuba or Djibouti or French Somaliland or Dominica or Dominican Republic or East Timor or East Timur or Timor Leste or Ecuador or Egypt or United Arab Republic or El Salvador or Eritrea or Ethiopia or Fiji or Gabon or Gabonese Republic or Gambia or Gaza or Georgia Republic or Georgian Republic or Ghana or Grenada or Guatemala or Guinea or Guiana or Guyana or Haiti or Honduras or India or Maldives or Indonesia or Iran or Iraq or Jamaica or Jordan or Kazakhstan or Kazakh or Kenya or Kiribati or Korea or Kosovo or Kyrgyzstan or Kirghizia or Kyrgyz Republic or Kirghiz or Kirgizstan or Lao PDR or Laos or Lebanon or Lesotho or Basutoland or Liberia or Libya or Macedonia or Madagascar or Malagasy Republic or Malaysia or Malaya or Malay or Sabah or Sarawak or Malawi or Mali or Marshall Islands or Mauritania or Mauritius or Agalega Islands or Mexico or Micronesia or Middle East or Moldova or Moldovia or Moldovian or Mongolia or Montenegro or Morocco or Ifni or Mozambique or Myanmar or Myanma or Burma or Namibia or Nepal or Netherlands Antilles or Nicaragua or Niger or Nigeria or Muscat or Pakistan or Palau or Palestine or Panama or Paraguay or Peru or Philippines or Philipines or Phillipines or Phillippines or Papua New Guinea or Romania or Rumania or Roumania or Rwanda or Ruanda or Saint Lucia or St Lucia or Saint Vincent or St Vincent or Grenadines or Samoa or Samoan Islands or Navigator Island or Navigator Islands or Sao Tome or Senegal or Serbia or Montenegro or Seychelles or Sierra Leone or Sri Lanka or Solomon Islands or Somalia or Sudan or Suriname or Surinam or Swaziland or South Africa or Syria or Tajikistan or Tadzhikistan or Tadjikistan or Tadzhik or Tanzania or Thailand or Togo or Togolese Republic or Tonga or Tunisia or Turkey or Turkmenistan or Turkmen or Uganda or Ukraine or Uzbekistan or Uzbek or Vanuatu or New Hebrides or Venezuela or Vietnam or Viet Nam or West Bank or Yemen or Zambia or Zimbabwe).ti,ab,kw. (2446958)
447 exp africa/ or exp africa, northern/ or algeria/ or egypt/ or libya/ or morocco/ or tunisia/ or exp “africa south of the sahara”/ or africa, central/ or cameroon/ or central african republic/ or chad/ or congo/ or “democratic republic of the congo”/ or equatorial guinea/ or gabon/ or africa, eastern/ or burundi/ or djibouti/ or eritrea/ or ethiopia/ or kenya/ or rwanda/ or somalia/ or south sudan/ or sudan/ or tanzania/ or uganda/ or africa, southern/ or angola/ or botswana/ or lesotho/ or malawi/ or mozambique/ or namibia/ or south africa/ or swaziland/ or zambia/ or zimbabwe/ or africa, western/ or benin/ or burkina faso/ or cape verde/ or cote d'ivoire/ or gambia/ or ghana/ or guinea/ or guinea‐bissau/ or liberia/ or mali/ or mauritania/ or niger/ or nigeria/ or senegal/ or sierra leone/ or togo/ or americas/ or exp caribbean region/ or exp west indies/ or exp central america/ or belize/ or costa rica/ or el salvador/ or guatemala/ or honduras/ or nicaragua/ or panama/ or panama canal zone/ or latin america/ or mexico/ or exp south america/ or argentina/ or bolivia/ or brazil/ or chile/ or colombia/ or ecuador/ or french guiana/ or guyana/ or paraguay/ or peru/ or suriname/ or uruguay/ or venezuela/ or asia/ or asia, central/ or kazakhstan/ or kyrgyzstan/ or tajikistan/ or turkmenistan/ or uzbekistan/ or exp asia, southeastern/ or borneo/ or brunei/ or cambodia/ or timor‐leste/ or indonesia/ or laos/ or malaysia/ or mekong valley/ or myanmar/ or philippines/ or singapore/ or thailand/ or vietnam/ or asia, western/ or bangladesh/ or bhutan/ or india/ or sikkim/ or middle east/ or afghanistan/ or bahrain/ or iran/ or iraq/ or israel/ or jordan/ or kuwait/ or lebanon/ or oman/ or qatar/ or saudi arabia/ or syria/ or turkey/ or united arab emirates/ or yemen/ or nepal/ or pakistan/ or sri lanka/ or far east/ or china/ or beijing/ or macau/ or tibet/ or korea/ or mongolia/ or taiwan/ or indian ocean islands/ or comoros/ or madagascar/ or mauritius/ or reunion/ or seychelles/ or pacific islands/ or exp melanesia/ or exp micronesia/ or polynesia/ or pitcairn island/ or exp samoa/ or tonga/ or prince edward island/ or west indies/ or “antigua and barbuda”/ or bahamas/ or barbados/ or cuba/ or dominica/ or dominican republic/ or grenada/ or guadeloupe/ or haiti/ or jamaica/ or martinique/ or netherlands antilles/ or puerto rico/ or “saint kitts and nevis”/ or saint lucia/ or “saint vincent and the grenadines”/ or “trinidad and tobago”/ or united states virgin islands/ or oceania/ (2424512)
448 ((developing or less* developed or under developed or underdeveloped or middle income or low* income or underserved or under served or deprived or poor*) adj (countr* or nation? or population? or region? or world or state*)).ti,ab,kw. (234706)
449 ((developing or less* developed or under developed or underdeveloped or middle income or low* income or poor*) adj econom*).ti,ab,kw. (2988)
450 (low* adj (gdp or gnp or gross domestic or gross national)).ti,ab,kw. (643)
451 (low adj3 middle adj3 countr*).ti,ab,kw. (30878)
452 (lmic or lmics or third world or lami countr*).ti,ab,kw. (15985)
453 transitional countr*.ti,ab,kw. (445)
454 or/444–453 (3687965)
455 443 and 454 (30919)
456 Women/ (8405706)
457 Pregnant Women/ (69649)
458 Female/ and (Adolescent/ or Adult/ or Young Adult/) (9600899)
459 (woman* or women* or matern* or mother* or wife or wives or pregnan*).ti,ab,kw. (4324318)
460 ((female? or girl*) adj5 (adolescen* or adult* or child‐bearing or childbearing or highschool* or school* or teen* or marry* or marriage* or marrie? or menstru* or post‐pubescen* or postpubescen* or post‐puberty or postpuberty or reproductive or spous* or youth* or young adult?)).ti,ab,kw. (284320)
461 ((female? or girl or girls) adj2 (ten year? or ten yr? or 10 year? or 10 yr? or eleven year? or eleven yr? or 11 year? or 11 yr? or twelve year? or twelve yr? or 12 year? or 12 yr?)).ti,ab,kw. (28338)
462 ((female? or girl or girls) adj2 (age? or old) adj3 (“10” or ten or “11” or eleven or “12” or twelve)).ti,ab,kw. (65402)
463 young girl?.ti,ab,kw. (9994)
464 or/456–463 (15074516)
465 455 and 464 (18238)
466 465 use coch,cctr,dare,clhta,cleed [COCHRANE RECORDS] (925)
467 114 or 244 or 357 or 466 [ALL DATABASES] (18543)
468 limit 467 to “yr=2016‐current” [Limit not valid in DARE; records were retained] (5061)
469 remove duplicates from 468 (3740)
470 limit 467 to “yr=2011–2015” [Limit not valid in DARE; records were retained] (5957)
471 remove duplicates from 470 (4296)
472 limit 467 to yr=“2000–2010” [Limit not valid in DARE; records were retained] (4760)
473 remove duplicates from 472 (3258)
474 467 not (468 or 470 or 472) (2767)
475 remove duplicates from 474 (2200)
476 469 or 471 or 473 or 475 [TOTAL UNIQUE RECORDS] (13492)
477 476 use medall [MEDLINE UNIQUE RECORDS] (6468)
478 476 use emczd [EMBASE UNIQUE RECORDS] (5659)
479 476 use medal, emczd, coch, cctr, dare, cleed, clhta (12605)
480 476 not 479 [PSYCINFO UNIQUE RECORDS] (887)
481 476 use coch [DSR UNIQUE RECORDS] (4)
482 476 use dare [DARE UNIQUE RECORDS] (1)
483 476 use cleed [NHS UNIQUE RECORDS] (0)
484 476 use clhta [HTA UNIQUE RECORDS] (0)
485 476 use CCTR [CENTRAL UNIQUE RECORDS] (473)

## APPENDIX C DATA EXTRACTION CODE BOOKS

Quantitative Study Codebook

Where possible, provide direct quotes and page numbers to support your extractions.

**Table:**

Code	Description
Study ID	From Covidence
Date coded	
Coder initials	
Correspondence required	Add a note here if authors will need to be contacted to request additional information
*Reference identification*	
Title	
First author	
Year	
Type of publication	(drop‐down) Journal article / Book / Conference abstract / Dissertation / Report / Other
Other	Define “other” publication type
Funder	
Country	
City and region (if applicable)	
Start date of study	
End date of study	
Study duration	
Aim of the study	
Authors’ definition of empowerment	
Authors’ rationale for empowerment approach	
*Methods*	
Study design	(drop‐down) RCT / cRCT / CBA / ITS / DID / RDD / PSM / other
Other	Define “other” study design type
Study design description	
Comparison group	Describe the nature of the comparison group
Statistical method	Describe method used and comment on appropriateness.
*Intervention*	
Number of treatment arms	(drop‐down) 1–10
Description of treatment arm 1	
Description of treatment arm 2 (continue adding as needed)	
Type of nutrition intervention	(drop‐down–select all that apply) Micronutrient supplementation / Fortification / Education or Counselling / School feeding / Other supplemental nutrition / Other
Description of nutrition intervention	
Type of agency intervention	(drop‐down–select all that apply) Mentorship program / Leadership skills training / Technical or occupational skills training / Program leadership role for girls / Peer education / Peer support group / Other
Description of agency intervention	
Type of opportunity structure intervention	(drop‐down—select all that apply) Cash transfer program / Savings and loan program / Education support / Girls’ rights advocacy or education (with parents, teachers, community influencers) / Prevention of early marriage and pregnancy / Other
Description of opportunity structure intervention	
Intervention setting	(drop‐down – select all that apply) School / Community / Home / Health Facility / Other
Other	Describe “other” intervention setting
Geography	(drop‐down – select all that apply) Urban / Rural / Remote / Slum / Other
Other	Describe other geographic setting
Intervention administrator	(drop‐down) Foreign government / local or national government / non‐government organization / community‐based organization / Academic institution / Other
Other	Describe other intervention administrator
Intervention provider	(drop‐down – select all that apply) Community health workers / Health facility staff / Teachers / Peers / Parents / Volunteers / Other
Other	Describe other intervention provider
Provider training	Describe the training of providers to deliver intervention
Recruitment	Describe procedures for recruiting participants
Attrition rate	The proportion of participants lost during the intervention or during follow‐up.
Reach	Describe the degree to which the intended audience participated in an intervention by “their presence”
Dose	Describe the proportion or amount of an intervention delivered to participants. E.g., measures of frequency, duration, and/or intensity
Fidelity / integrity	Describe the degree to which the intervention was delivered as intended.
Adaptation	Describe the degree to which the program content was intentionally changed during implementation to improve programme effectiveness
Prior needs assessment	Describe any needs assessment that was conducted to inform intervention design
Reminders	Describe any prompts or reminders sent to participants to attend / participate in intervention
Quality	Describe any findings related to the quality of quality of intervention materials/ resources (e.g., curriculum, training, and policy)
Cultural appropriateness	Describe any findings related to the cultural appropriateness of the intervention
Contamination	Describe any unintentional delivery of intervention to the control group or inadvertent failure to deliver intervention to experimental group
Cointervention	Describe any instances where interventions other than the treatment were applied differently to intervention conditions
Participant engagement	Describe participant's interaction with or receptivity to the intervention (i.e., what they think or how they feel about the intervention)
Contextual factors	Describe any social, built, and political factors internal (e.g., partnerships) and external to the intervention environment (e.g., social norms) that shape implementation.
Study population	
Number of participants	
Number in intervention group	
Number in comparison group	
Age range of study participants	Enter age range of study target population, e.g. 15–49 years
Age range of adolescent study participants	Enter age range for female adolescents who are included in study, e.g., 15–24 years
Age sub‐groups	List all age sub‐groups for which data are provided, e.g. study covers 15–49 years, and analyses were done separately for 15–19 years, 20–24 years, 25–49 years
Did the study population also include men or boys?	(drop‐down) Yes / No / Unclear
Adolescent participants’ school status	(drop‐down) In school / out of school / Both / Unclear
Race / ethnicity / language	Describe
Religion	Describe
Socio‐economic status	Describe
Disability, physical health	Describe
Includes pregnant participants	(drop‐down) Yes / No / Unclear
Includes young mothers	(drop‐down) Yes / No / Unclear
Conflict setting	(drop‐down) Yes / No / Unclear
Food insecure setting	(drop‐down) Yes / No / Unclear
*Outcomes*	
Primary outcomes	Define outcomes and how they are measured
Secondary outcomes	Define outcomes and how they are measured
Method of assessing outcomes	Describe methods for assessing outcome measures
Validity and reliability of outcomes measures	Describe primary authors’ views on the validity and reliability of outcome measures
Follow‐up of non‐respondents	Describe the methods used to follow‐up non‐respondents
Timing of outcome assessment	Describe frequency of assessment, length of follow‐up, etc.
*Results*	
Number of participants in Treatment Arm 1 (continue adding rows for each treatment arm)	
Number of participants in Treatment Arm 2 (continue adding rows for each treatment arm)	
Number of participants in comparison group	
*For each outcome…*	
Direction of effect	(drop‐down) Effect favours treatment / Effect favours comparison / Effect favours neither / Cannot tell
Effect statistically significant?	(drop‐down) Yes / No / Cannot tell
Treatment sample size	
Control sample size	
Number of missing participants	
Nature of the measure	(drop‐down) Continuous / Dichotomous / Other
*If outcome measure is continuous…*	
Treatment group mean	
Comparison group mean	
Are reported means adjusted?	(drop‐down) Yes / No / Cannot tell
Treatment group standard deviation	
Comparison group standard deviation	
Treatment group standard error	
Comparison group standard error	
*t*‐value for an independent t‐test	
*If outcome measure is dichotomous…*	
Treatment group number of participants who experienced a change	
Comparison group number of participants who experienced a change	
Treatment group proportion of participants who experienced a change	
Comparison group proportion of participants who experienced a change	
Are the proportions adjusted for pretest variables?	
Logged odds ratio	
Standard error of logged odds ratio	
Adjusted logged odds ratio	
Chi‐square with degrees of freedom	
Correlation coefficient	
*Adverse events*	
Adverse events	List all adverse events of integrating women's empowerment, as described by primary authors.

Qualitative study codebook.

**Table:**

Code	Description
Study ID	from Covidence
Date coded	
Coder initials	
Correspondence required	Add a note here if authors will need to be contacted to request additional information

*Reference identification*	
Title	
First author	
Year	
Type of publication	(drop‐down) Journal article / Book / Conference abstract / Dissertation / Report / Other
Other	Define “other” publication type
Funder	
Country	
City and region (if applicable)	
Start date of study	
End date of study	
Aim of the study	
Authors’ definition of empowerment	
Authors’ rationale for empowerment approach	
	
*Methods*	
Study design description	
Study duration	
Participant inclusion/exclusion criteria	
*Quality assessment (CASP)*	
Was there a clear statement of the aims of the research?	(drop‐down) Yes / No / Unclear
Support for judgement (incl. pg #)	
Is a qualitative methodology appropriate?	(drop‐down) Yes / No / Unclear
Support for judgement (incl. pg #)	
Was the research design appropriate to address the aims of the research?	(drop‐down) Yes / No / Unclear
Support for judgement (incl. pg #)	
Was the recruitment strategy appropriate to the aims of the research?	(drop‐down) Yes / No / Unclear
Support for judgement (incl. pg #)	
Was the data collected in a way that addressed the research issue?	(drop‐down) Yes / No / Unclear
Support for judgement (incl. pg #)	
Has the relationship between researcher and participants been adequately considered?	(drop‐down) Yes / No / Unclear
Support for judgement (incl. pg #)	
Have ethical issues been taken into consideration?	(drop‐down) Yes / No / Unclear
Support for judgement (incl. pg #)	
Was the data analysis sufficiently rigorous?	(drop‐down) Yes / No / Unclear
Support for judgement (incl. pg #)	
Is there a clear statement of findings?	(drop‐down) Yes / No / Unclear
Support for judgement (incl. pg #)	
How valuable is the research?	(drop‐down) Yes / No / Unclear
Support for judgement (incl. pg #)	
	
*Study population*	
Number of participants	
Study scale	Small (e.g., one/several village) / large (e.g., district, region, province)
Describe scale	
Study setting	(drop‐down) Urban / Rural / Informal‐urban / Remote / Other
Describe setting	
Target	(drop‐down) Individual / Household / Community / Other
Describe target	
Age range of study participants	Enter age range of study target population, e.g. 15–49 years
Adolescent age range	Enter age range for female adolescents who are included in study, e.g., 15–24 years
Age subgroups	List all female age sub‐groups for which data are provided, e.g. study covers 15–49 years, and analyses were done separately for 15–19 years, 20–24 years, 25–49 years
Did the study population also include men or boys?	(drop‐down) Yes / No / Unclear
Adolescent participants’ school status	(drop‐down) In school / out of school / Both / Unclear
Race / ethnicity / language	Describe
Religion	Describe
Socio‐economic status	Describe
Disability, physical health	Describe
Includes pregnant participants	(drop‐down) Yes / No / Unclear
Includes young mothers	(drop‐down) Yes / No / Unclear
Conflict setting	(drop‐down) Yes / No / Unclear
Food insecure setting	(drop‐down) Yes / No / Unclear
	
*Intervention*	
Type of nutrition intervention	(drop‐down – select all that apply) Micronutrient supplementation / Fortification / Education or Counselling / School feeding / Other supplemental nutrition / Other
Description of nutrition intervention	
Type of agency intervention	(drop‐down – select all that apply) Mentorship program / Leadership skills training / Technical or occupational skills training / Program leadership role for girls / Peer education / Peer support group / Other
Description of agency intervention	
Type of opportunity structure intervention	(drop‐down – select all that apply) Cash transfer program / Savings and loan program / Education support / Girls’ rights advocacy or education (with parents, teachers, community influencers) / Prevention of early marriage and pregnancy / Other
Description of opportunity structure intervention	
Intervention setting	(drop‐down – select all that apply) School / Community / Home / Health Facility / Other
Describe intervention setting	
Intervention administrator	(drop‐down) Foreign government / local or national government / non‐government organization / community‐based organization / Academic institution / Other
Describe intervention administrator	
Intervention provider	(drop‐down – select all that apply) Community health workers / Health facility staff / Teachers / Peers / Parents / Volunteers / Other
Describe intervention provider	
Provider training	Describe the training of providers to deliver intervention
Recruitment	Describe procedures for recruiting participants
Attrition rate	The proportion of participants lost during the intervention or during follow‐up.
Reach	Describe the degree to which the intended audience participated in an intervention by “their presence”
Dose	Describe the proportion or amount of an intervention delivered to participants. E.g., measures of frequency, duration, and/or intensity
Fidelity / integrity	Describe the degree to which the intervention was delivered as intended.
Adaptation	Describe the degree to which the program content was intentionally changed during implementation to improve programme effectiveness
Prior needs assessment	Describe any needs assessment that was conducted to inform intervention design
Reminders	Describe any prompts or reminders sent to participants to attend / participate in intervention
Quality	Describe any findings related to the quality of quality of intervention materials/ resources (e.g., curriculum, training, and policy)
Cultural appropriateness	Describe any findings related to the cultural appropriateness of the intervention
Contamination	Describe any unintentional delivery of intervention to the control group or inadvertent failure to deliver intervention to experimental group
Cointervention	Describe any instances where interventions other than the treatment were applied differently to intervention conditions
Participant engagement	Describe participant's interaction with or receptivity to the intervention (i.e., what they think or how they feel about the intervention)
Contextual factors	Describe any social, built, and political factors internal (e.g., partnerships) and external to the intervention environment (e.g., social norms) that shape implementation.
Measures	
*Feasibility (the extent to which an intervention is practical or viable in a particular context or situation)*	
Cultural sensitivity of program	
Participant engagement	
Community/public commitment	
*Appropriateness (the extent to which an intervention or activity fits with a particular context or situation)*	
Participants’ views on programme acceptability	
Implementers’ views on programme acceptability	
Community support for programme	
*Meaningfulness (the extent to which an intervention or activity is positively experienced by an individual or group)*	
Participants’ views on programme as a positive or negative experience	
Participants’ views on benefits and costs of participation	
Participants’ awareness of own nutritional needs	
Participants’ motivation to act for improved nutrition	
Community desire to support participants to act for improved nutrition	
*Adverse events*	
Adverse events	List all adverse events of integrating women's empowerment, as described by primary authors.
